# Outer membrane vesicles and the outer membrane protein OmpU govern *Vibrio cholerae* biofilm matrix assembly

**DOI:** 10.1128/mbio.03304-23

**Published:** 2024-01-11

**Authors:** Anna Potapova, William Garvey, Peter Dahl, Shuaiqi Guo, Yunjie Chang, Carmen Schwechheimer, Michael A. Trebino, Kyle A. Floyd, Brett S. Phinney, Jun Liu, Nikhil S. Malvankar, Fitnat H. Yildiz

**Affiliations:** 1 Department of Microbiology and Environmental Toxicology, University of California-Santa Cruz, Santa Cruz, California, USA; 2 Department of Molecular Biophysics and Biochemistry, Yale University, New Haven, Connecticut, USA; 3 Microbial Sciences Institute, Yale University, West Haven, Connecticut, USA; 4 Department of Microbial Pathogenesis, Yale School of Medicine, New Haven, Connecticut, USA; 5 Proteomics Core Facility, UC Davis Genome Center, University of California-Davis, Davis, California, USA; University of Michigan-Ann Arbor, Ann Arbor, Michigan, USA

**Keywords:** *Vibrio cholerae*, biofilms, biofilm matrix, outer membrane proteins

## Abstract

Biofilms are matrix-encased microbial communities that increase the environmental fitness and infectivity of many human pathogens including *Vibrio cholerae*. Biofilm matrix assembly is essential for biofilm formation and function. Known components of the *V. cholerae* biofilm matrix are the polysaccharide *Vibrio* polysaccharide (VPS), matrix proteins RbmA, RbmC, Bap1, and extracellular DNA, but the majority of the protein composition is uncharacterized. This study comprehensively analyzed the biofilm matrix proteome and revealed the presence of outer membrane proteins (OMPs). Outer membrane vesicles (OMVs) were also present in the *V. cholerae* biofilm matrix and were associated with OMPs and many biofilm matrix proteins suggesting that they participate in biofilm matrix assembly. Consistent with this, OMVs had the capability to alter biofilm structural properties depending on their composition. OmpU was the most prevalent OMP in the matrix, and its absence altered biofilm architecture by increasing VPS production. Single-cell force spectroscopy revealed that proteins critical for biofilm formation, OmpU, the matrix proteins RbmA, RbmC, Bap1, and VPS contribute to cell-surface adhesion forces at differing efficiency, with VPS showing the highest efficiency whereas Bap1 showing the lowest efficiency. Our findings provide new insights into the molecular mechanisms underlying biofilm matrix assembly in *V. cholerae*, which may provide new opportunities to develop inhibitors that specifically alter biofilm matrix properties and, thus, affect either the environmental survival or pathogenesis of *V. cholerae*.

**IMPORTANCE:**

Cholera remains a major public health concern. *Vibrio cholerae*, the causative agent of cholera, forms biofilms, which are critical for its transmission, infectivity, and environmental persistence. While we know that the *V. cholerae* biofilm matrix contains exopolysaccharide, matrix proteins, and extracellular DNA, we do not have a comprehensive understanding of the majority of biofilm matrix components. Here, we discover outer membrane vesicles (OMVs) within the biofilm matrix of *V. cholerae*. Proteomic analysis of the matrix and matrix-associated OMVs showed that OMVs carry key matrix proteins and *Vibrio* polysaccharide (VPS) to help build biofilms. We also characterize the role of the highly abundant outer membrane protein OmpU in biofilm formation and show that it impacts biofilm architecture in a VPS-dependent manner. Understanding *V. cholerae* biofilm formation is important for developing a better prevention and treatment strategy framework.

## INTRODUCTION

Biofilms are communities of microbial aggregates surrounded by an extracellular matrix. They are present in diverse environments and represent a survival strategy for microorganisms (1, 2). Biofilm development and function depend on the biofilm matrix, which enables microorganisms to grow and survive in a wide range of conditions (3, 4). Biofilm matrix composition depends on the microorganism(s), environmental conditions, and biofilm growth stage but typically includes polysaccharides, proteins, lipids, and nucleic acids (1, 5). Other components include outer membrane vesicles (OMVs), which are spherical structures released from the outer membrane of Gram-negative bacteria (6). The biofilm matrix facilitates surface attachment, provides structural support, mediates microbial interactions, degrades nutrients, and is a barrier to environmental stresses (7–9). Thus, understanding the composition and properties of the biofilm matrix and the mechanisms of biofilm matrix assembly can help develop biofilm prevention and microbial control strategies.

*Vibrio cholerae* is a Gram-negative bacterium that causes the acute diarrheal disease cholera, which annually affects millions worldwide (10, 11). Biofilm formation is essential for the pathogen’s environmental persistence, transmission to humans, and host infectivity (12–18). *V. cholerae* biofilm formation begins when the bacterium attaches to a surface and forms a biofilm matrix, which enables the formation of microcolonies and mature biofilms (4, 19–22). A major component of the *V. cholerae* biofilm matrix is the *Vibrio* polysaccharide (VPS), which is essential for matrix formation and stabilization (23–25). In some strains of *V. cholerae,* extracellular DNA (eDNA) is present in the biofilm matrix, which interacts with VPS contributing to biofilm structural strength (26). The major components of the *V. cholerae* biofilm matrix are proteins, some of which have been well characterized. For example, RbmA, Bap1, and RbmC have complementary roles in biofilm formation by promoting cell-surface attachment, stabilizing founder cell-daughter cell interactions, and promoting matrix assembly by interacting with VPS (27–31).

OMVs are produced by Gram-negative bacteria either through blebbing of the outer membrane (OM) or explosive cell lysis (32, 33). *V. cholerae* also produces OMVs during its growth and deciphering the mechanism and regulation of OMV formation in *V. cholerae* is an emerging area (34–36). The proposed phospholipid transporter VacJ/Yrb ABC (ATP-binding cassette) transport system has been linked to OMV formation by *V. cholerae*: the absence of Yrb increases OMV production without compromising OM integrity (35). The small non-coding RNA, VrrA (Vibrio regulatory RNA of *ompA*), of *V. cholera*e positively controls OMV release by downregulating the outer membrane protein (OMP) OmpA: overexpression of VrrA and deletion of *ompA* increase OMV production (36). When *V. cholerae* is grown under virulence-inducing conditions, it makes OMVs that deliver bioactive cholera toxin to intestinal epithelial cells and protect the OMV-attached toxin from being degraded in the intestine (37, 38). Increased OMV production changes the cell surface, which helps *V. cholerae* adapt to the microenvironment of the host. OMVs are immunogenic, and immunizing adult female mice with OMVs protects their nursing offspring from being colonized by *V. cholerae* (39). Furthermore, *V. cholerae* OMVs reduce phage predation, thus serving as a defense mechanism (40). The protein content of OMVs made by *V. cholerae* grown under virulence-inducing conditions is comprised of OMPs, periplasmic proteins, biofilm matrix proteins, and other proteins secreted by the type II secretion system. Additionally, solid-state NMR and initial proteome studies of the *V. cholerae* biofilm matrix showed the presence of phospholipids and outer membrane proteins (41, 42). However, we lack a full understanding of the *V. cholerae* matrix proteome, including whether OMVs are present and if they play a role in biofilm assembly and structural integrity.

In this study, we present the *V. cholerae* biofilm matrix proteome, show that OMVs are, indeed, present in the matrix, and describe the cargo carried by OMVs. We show that the major OMP, OmpU, affects biofilm formation. We systematically analyze the impact of OmpU on the production and localization of key matrix components and reveal that it predominantly impacts VPS production. Together, OmpU, key matrix proteins, and VPS contribute to the cell-cell and cell-surface adhesion at the early stages of biofilm formation. OMVs contribute to biofilm matrix assembly by carrying key matrix proteins and VPS. This study provides critical insights into *V. cholerae* biofilm matrix assembly and furthers our understanding of the mechanisms of an important cellular process contributing to the *V. cholerae* infection cycle.

## RESULTS

### *V. cholerae* biofilm matrix is enriched in outer membrane proteins and outer membrane vesicles

**Fig 1:**
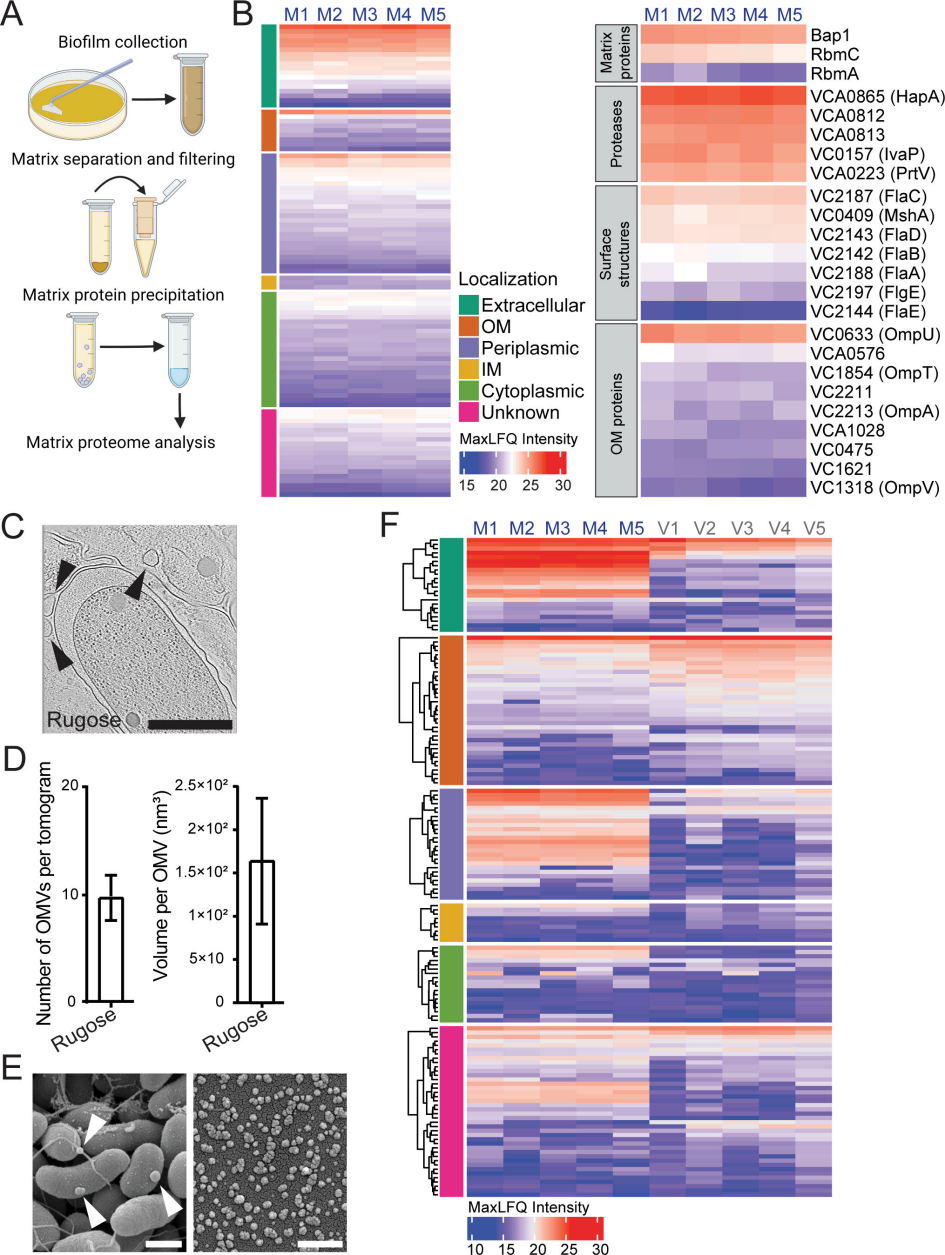
The *V. cholerae* biofilm matrix is enriched in outer membrane proteins and outer membrane vesicles. (**A**) Schematic representation of the biofilm matrix proteome analysis experimental design. Created with BioRender.com. (**B**) Proteome data were analyzed using Proteome Discoverer software; five biological replicates were analyzed, and proteins found in at least two of the samples were included in further analysis. Heatmap view of the abundant proteins in the matrix proteome. Each column indicates a biological replicate, and each row indicates a single protein found in the biofilm matrix (left heatmap). Proteins on the left heatmap are clustered based on the subcellular localization predicted by the PSortB online tool (44). Corresponding subcellular localizations are indicated in the legend, and color scale represents relative protein abundance. Functional categories of a set of biofilm matrix proteins are shown in the right heatmap. MaxLFQ values represent protein abundance. (**C**) Representative snapshots from cryo-ET tomograms depicting the interior of a biofilm matrix of biofilm grown cells of *V. cholerae*. Arrows point to the OMVs. Scale bars = 0.5 µm. (**D**) Average number of OMVs and volume per OMV found in the tomograms (*n* = 10) of *V. cholerae*. Error bars represent the standard error of the mean. (**E**) Representative scanning electron microscopy images of the biofilm grown cells with associated OMVs and OMVs isolated from biofilm grown cells. Arrows point to the OMVs. Scale bars = 0.5 µm. (**F**) Heatmap view of the proteins found in biofilm matrix (**M1–M5**) and matrix OMV proteomes (**V1–V5**). Each column indicates a biological replicate, and each row indicates a single protein. Predicted subcellular localizations are indicated in the legend, and MaxLFQ values represent protein abundance. For each given subcellular localization group, proteins were clustered hierarchically.

The *V. cholerae* biofilm matrix proteome has not been comprehensively characterized. Therefore, we determined the *V. cholerae* biofilm matrix composition in a biofilm-overproducing rugose variant of *V. cholerae* O1 El Tor strain A1552 (25, 28, 43). The biofilm matrix from biofilms grown on membrane filters placed on a nutrient agar plate was separated from cells, and biofilm matrix proteins were precipitated. The resulting proteome was analyzed by liquid chromatography-tandem mass spectrometry (LC-MS/MS) (Fig. 1A). The full list of analyzed proteins is provided in Table S1.

Four hundred fourty-nine proteins were present in the matrix proteome including known matrix proteins RbmA, RbmC, and Bap1, consistent with the expected localization of these proteins. We focused on the 100 most abundant proteins and first predicted their subcellular localization using the PSORTb online subcellular localization prediction tool (Fig. 1B). Fifty-three proteins (87% of the spectrum counts) were predicted to be extracellular, outer membrane, and periplasmic, 28 proteins to be inner membrane and cytoplasmic (6% of the spectrum counts), and 19 proteins were of unknown localization (7% of the spectrum counts).

In the matrix proteome, we detected pilins (MshA) and flagellins, which are protein components of cell surface structures. We also found an enrichment of periplasmic proteins and outer membrane proteins (OMPs) (Fig. 1B), prompting us to evaluate whether outer membrane vesicles are components of the *V. cholerae* biofilm matrix. Using cryo-focused-ion-beam (cryo-FIB) milling and cryo-electron tomography (cryo-ET) (Fig. S1), we visualized OMVs within the biofilm matrix and quantified OMV production by measuring the number of OMVs per tomogram, and OMV volumes; we found that biofilm matrix of the rugose strain contained vesicles with the average volume of 1.5 × 10^2^ nm^3^ (Fig. 1C and D). Taken together, these findings show that the *V. cholerae* biofilm matrix harbors OMVs and suggest that OMPs found in the biofilm matrix (Fig. 1B) are derived from OMVs.

To further investigate the contribution of OMVs to the matrix proteome, we isolated OMVs from the biofilm matrix using density gradient ultracentrifugation. Scanning EM of biofilm-associated OMVs and isolated OMVs are shown in Fig. 1E. We determined the OMV proteome using LC-MS/MS. One hundred ninety-four proteins were present within the OMV proteome, and 150 of them were also found within the biofilm matrix (Table S2). This indicates that 33% of the total matrix proteome is contributed through OMVs.

Ninety-nine of these OMV proteins (82% of the spectrum counts) were predicted to be extracellular, outer membrane, and periplasmic, 37 proteins were inner membrane and cytoplasmic (2% of the spectrum counts), and 58 proteins were of unknown location (16% of the spectrum counts).

In particular, we found the matrix proteins Bap1, RbmC, and RbmA, as well as proteases HapA and PrtV present in OMVs and the matrix (Fig. 1F). As expected, all the OMPs found in the biofilm matrix were enriched on the OMVs. By measuring the OmpU abundance in biofilm matrix in total biofilms, cell-free biofilm matrix, OMV-free biofilm matrix, OMVs, we evaluated the origin of OmpU in biofilms. We found that OmpU in biofilm matrix originates predominantly from OMVs (Fig. S2). Collectively, our results show that the *V. cholerae* matrix proteome is enriched in OMVs and that major matrix proteins are associated with OMVs in the matrix.

### The outer membrane protein OmpU affects biofilm formation

**Fig 2:**
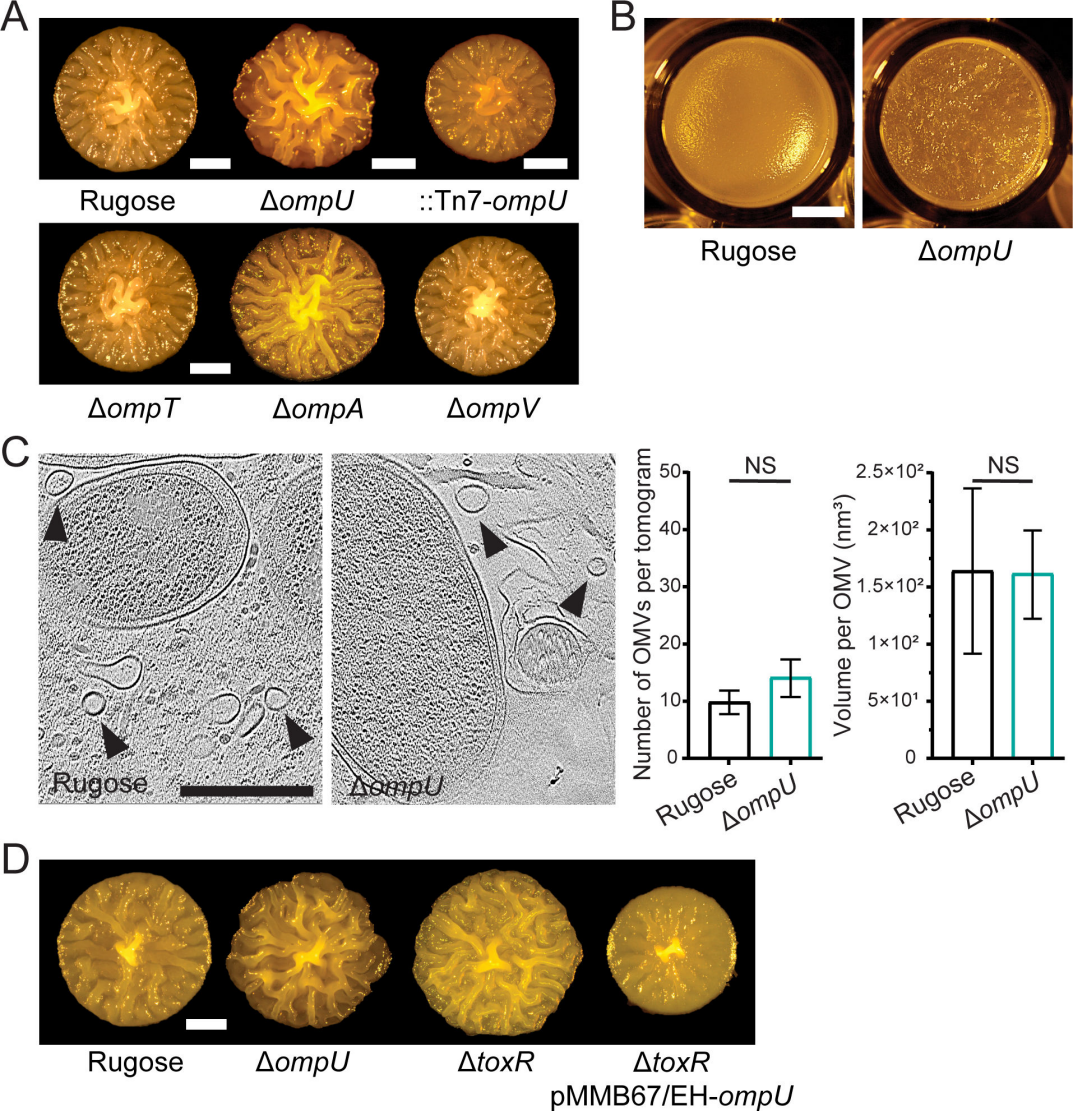
The outer membrane porin OmpU affects biofilm formation. (**A, D**) Colony corrugation phenotypes of strains with indicated genotypes after 72 h of growth at 30°C. Images are representative of two biological replicates, with three technical replicates per biological replicate. Scale bars = 1 mm. (**B**) Morphology of pellicles formed after 24 h incubation at 30°C. Scale bars = 2 mm. (**C**) Representative cryo-ET tomograms depicting the interior of a biofilm matrix of biofilm grown rugose and Δ*ompU* cells of *V. cholerae*. Arrows point to the OMVs. Scale bars = 0.5 µm. Average number of OMVs (left) and volume per OMV (right) found in the tomograms (*n* = 10) of *V. cholerae* biofilms for the rugose and Δ*ompU* strains, respectively. Error bars represent the standard error of the mean, and statistical significance was determined using Student’s *t*-test. NS, not significant.

To understand the role of OMPs in biofilm formation, we focused on the four OMPs (OmpU, OmpT, OmpA, and OmpV) that were present in both matrix and OMV proteomes. We generated in-frame deletions of *ompU*, *ompT, ompA,* and *ompV* and first analyzed their potential roles in colony biofilm architecture. The strain lacking *ompU* showed markedly increased colony corrugation which indicates an increase in biofilm matrix production or changes to biofilm matrix assembly; this phenotype of Δ*ompU* was complemented by introducing the wild-type copy of the *ompU* gene under the control of the native promoter at the neutral Tn7 site on the chromosome. Colony corrugation phenotypes of the Δ*ompT*, Δ*ompA,* and Δ*ompV* were similar to that of the rugose strain (Fig. 2A).

Since Δ*ompU* showed the most pronounced biofilm phenotype, we focused our study on OmpU. We evaluated pellicle formation, biofilms formed at air-liquid interfaces, and found that Δ*ompU* pellicles showed enhanced corrugation compared to rugose pellicles (Fig. 2B), suggesting that Δ*ompU* has enhanced biofilm forming ability. We next analyzed OMV production in Δ*ompU*. We found that OMV production, as evaluated by the number of OMVs produced and OMV volumes, were similar to the rugose parental strain (Fig. 2C), suggesting that increased biofilm formation is not due to altered OMV formation.

OmpU is one of the most highly expressed OMPs in *V. cholerae*. Its expression is positively regulated by ToxR, the main transcriptional regulator of the *V. cholerae* virulence regulatory circuitry (45–47). Thus, we generated a Δ*toxR* strain and compared its colony corrugation phenotype to the Δ*ompU* strain. We found that Δ*toxR* and Δ*ompU* phenocopied each other and expression of *ompU* in-trans in the Δ*toxR* strain reduced colony corrugation (Fig. 2D). This result supports the role of ToxR in biofilm formation through regulation of OmpU.

### OmpU differentially impacts the abundance of biofilm matrix components

**Fig 3:**
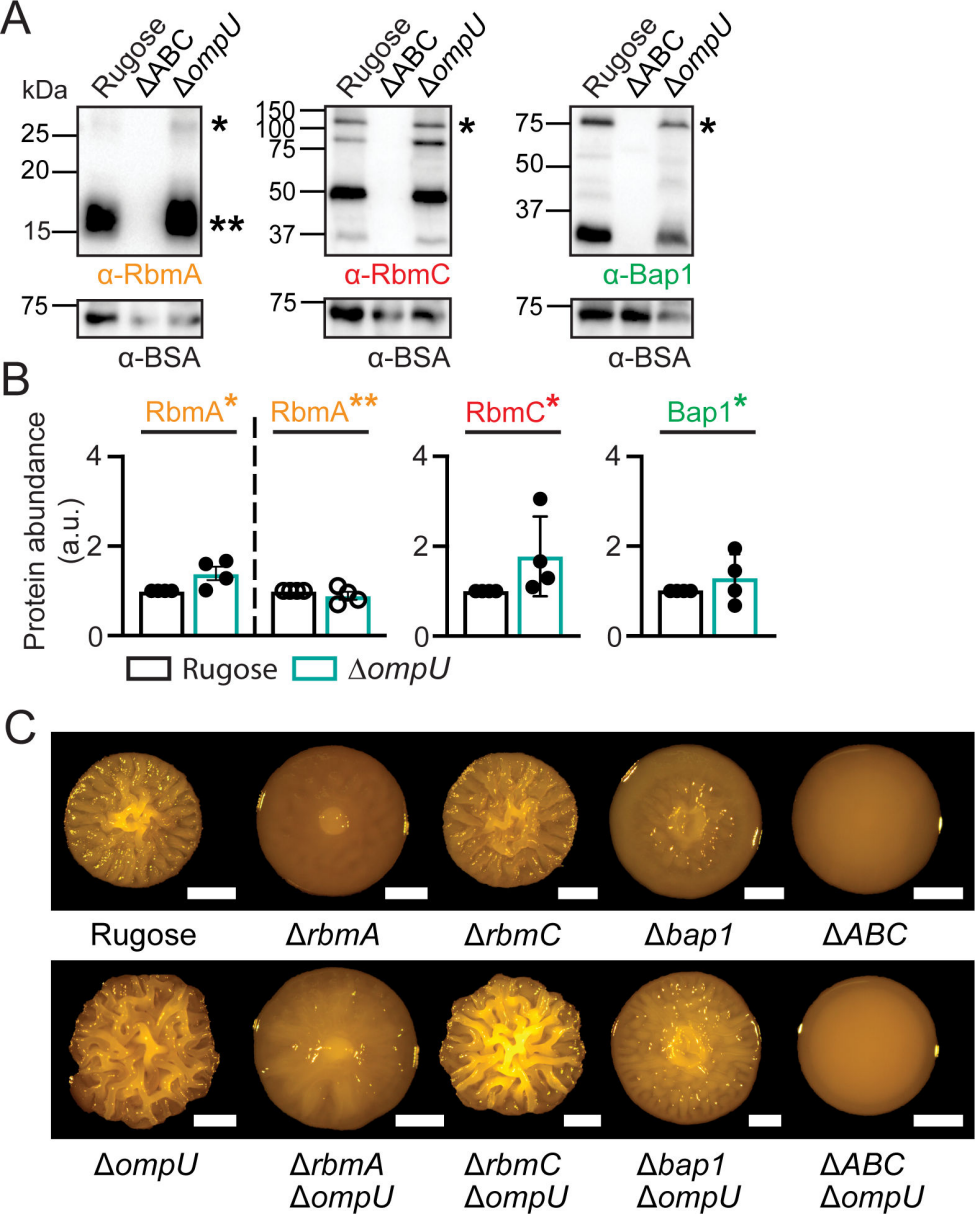
OmpU does not change matrix protein abundance and affects biofilm colony architecture independently of RbmA, RbmC, and Bap1. (**A**) Representative immunoblots showing biofilm matrix proteins. Blots were probed against RbmA (left), RbmC (middle), and Bap1 (right) antibodies; single asterisk (*) indicates full-length protein [predicted sizes are 32.5 kDa (RbmA), 114.8 kDa (RbmC), and 82.9 (Bap1)], while double asterisk (**) indicates a proteolytically cleaved variant of RbmA (predicted size 22 kDa). ΔABC stands for the triple *rbmA rbmC bap1* deletion strain. Bovine serum albumin (BSA) was added to each sample at equal concentrations, and blots were probed with BSA (predicted size 66.5 kDa) antibodies. Protein ladder sizes in kDa are indicated on the left side of each immunoblot. (**B**) Analysis of the abundance of the full-length and processed matrix proteins in the biofilm matrix of rugose Δ*ompU* strains. Protein accumulation was analyzed using ImageJ-Fiji software based on at least two biological replicates with two technical replicates per biological replicate. Error bars represent the standard error of the mean. (**C**) Colony corrugation phenotypes of strains with indicated genotypes after 72 h of growth at 30°C. Images are representative of two biological replicates, with three technical replicates per biological replicate. Scale bars = 1 mm.

Colony biofilm architecture relies on the production of matrix proteins and VPS. Therefore, we compared the abundance of matrix proteins RbmA, RbmC, and Bap1 between rugose and Δ*ompU* strains, with a Δ*rbmA* Δ*rbmC* Δ*bap1* (ΔABC) strain as the negative control. We isolated the biofilm matrix from these strains and performed immunoblot analysis (Fig. 3A). Full-length RbmA and its proteolytically cleaved variant, as well as full-length RbmC and Bap1 accumulated at similar levels in the rugose and Δ*ompU* biofilms (Fig. 3B). We also observed that degradation products of RbmC and Bap1 accumulated similarly between rugose and Δ*ompU* strains (Fig. 3A). Thus, the increased colony corrugation seen in the Δ*ompU* strain was not caused by differences in RbmA, RbmC, or Bap1 levels.

To evaluate how OmpU contributes to colony biofilm architecture and if it is in the same genetic pathway with *rbmA*, *rbmC,* and *bap1*, we generated the following double deletion strains: Δ*ompU*Δ*rbmA*, Δ*ompU*Δ*rbmC*, and Δ*ompU*Δ*bap1*, as well as the quadruple Δ*ompU*ΔABC strain. Colony corrugation analysis showed that each double deletion strain exhibited a colony corrugation level intermediate of those of the single mutants. Both the triple Δ*rbmA* Δ*rbmC* Δ*bap1* and the quadruple deletion strains had a smooth colony morphology; however, the colony compactness/size of the Δ*ompU*ΔABC quadruple mutant was higher than that of the ΔABC triple mutant (Fig. 3C). Collectively, these results show that OmpU acts independently of each single matrix protein in establishing biofilm architecture.

**Fig 4:**
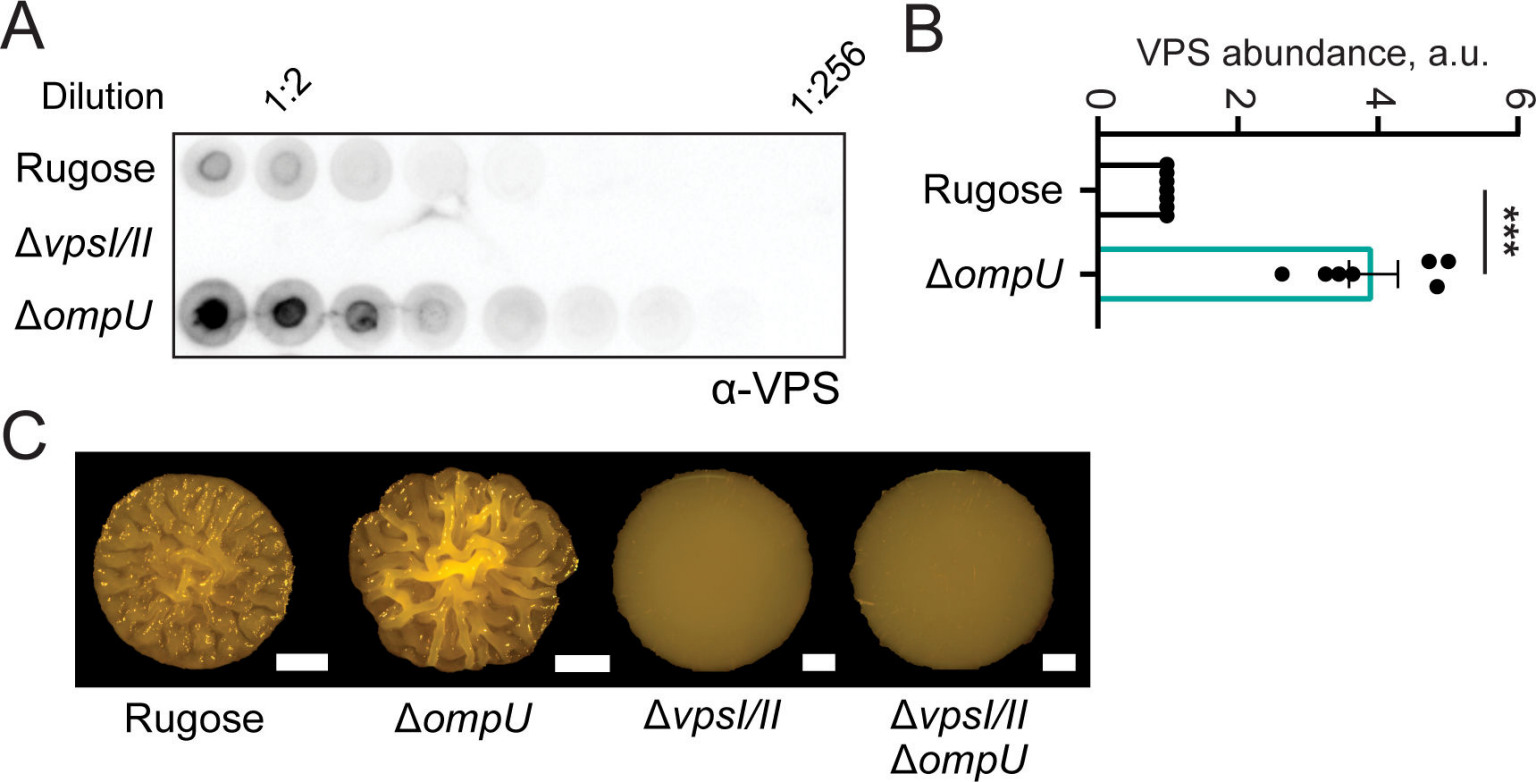
Strains lacking OmpU have increased VPS production. (**A**) Representative VPS dot-blot showing serial dilutions of VPS samples obtained from biofilms of indicated strains. Biofilms were grown for 24 h at 30°C on LB agar plates, and VPS was isolated as described in the Materials and Methods. The immunoblot was probed with VPS-specific antibodies. (**B**) Relative VPS amounts were calculated based on VPS immunoblots. The experiment was performed with three biological replicates and two technical replicates per each biological replicate. Immunoblots were analyzed using ImageJ-Fiji software, and statistical significance was determined using Student’s *t*-test. ***P ˂ 0.0001. Error bars represent the standard error of the mean. (**C**) Colony corrugation phenotypes of strains with indicated genotypes after 72 h of growth at 30°C. Images are representative of two biological replicates, with three technical replicates per biological replicate. Scale bars = 1 mm.

We also compared the abundance of VPS between the rugose and Δ*ompU* strains, with a strain lacking the two operons encoding the VPS production machinery (Δ*vpsI*Δ*vpsII*) as a negative control. We isolated VPS from the biofilm matrix of these strains and performed immunoblot analysis (Fig. 4A). VPS abundance was significantly higher in the Δ*ompU* strain (Fig. 4B). This finding indicates that OmpU controls VPS accumulation during biofilm formation. We also compared the colony corrugation phenotype of Δ*vpsI*Δ*vpsII* and Δ*vpsI*Δ*vpsII*Δ*ompU* strains. As expected, the Δ*vpsI*Δ*vpsII*Δ*ompU* strain phenocopied the Δ*vpsI*Δ*vpsII* mutant, validating that VPS is required for the increased corrugation observed in the Δ*ompU* strain (Fig. 4C).

Increased VPS production in the Δ*ompU* strain (Fig. 4A and B) prompted us to investigate if the lack of OmpU alters a signal transduction pathway(s) and thereby more generally affects the cell’s proteome profile, or if it has a more selective effect on specific proteins. Therefore, we compared whole-cell proteomes of rugose and Δ*ompU* strains by quantitative proteomics using tandem mass tag (TMT)-labeling coupled with LC-MS/MS. We analyzed only proteins showing at least twofold difference in abundance and found that lack of OmpU caused a significant difference in the abundance of a small set of proteins, where 27 proteins showed an increase and 8 proteins a decrease in abundance upon loss of OmpU. Table S3 contains the full list of proteins identified.

We detected an increase in the levels of VpsM, a protein necessary for VPS production, and VpsT, a positive transcriptional regulator of *vps* operons consistent with the observed increase in VPS production in the Δ*ompU* strain (Fig. S3A).

Three OMPs (OmpT, OmpA, and OmpV), two extracellular proteases (PrtV and IvaP), and two uncharacterized hypothetical proteins (VC2662 and VCA0713) were also enriched in the Δ*ompU* proteome. To determine if the increase in abundance of VC2662 and VCA0713 was responsible for the colony corrugation of the Δ*ompU* strain, we generated Δ*ompU* ΔVC2662 and Δ*ompU* ΔVCA0713 double mutants. We found that phenotypes of these strains were similar to the Δ*ompU* strain (Fig. S3B). Taken together, lack of OmpU does not appear to alter major signal transduction pathways or overall cellular proteome composition under the conditions used in this study.

### OmpU, matrix proteins, and VPS contribute differentially to the adhesion forces of cells in biofilms

Formation of robust biofilms requires the ability of cells to adhere to surfaces and to each other. The VPS and VPS-binding proteins RbmA, RbmC, and Bap1 are critical to the development of biofilm architecture and have the ability to bind VPS, suggesting that these proteins collectively support biofilm adhesion (28–31). The individual roles of these matrix proteins and the connection between a protein’s contribution to biofilm adhesion, as well as the role of biofilm adhesion in the development of biofilm architecture, are yet to be resolved.

**Fig 5:**
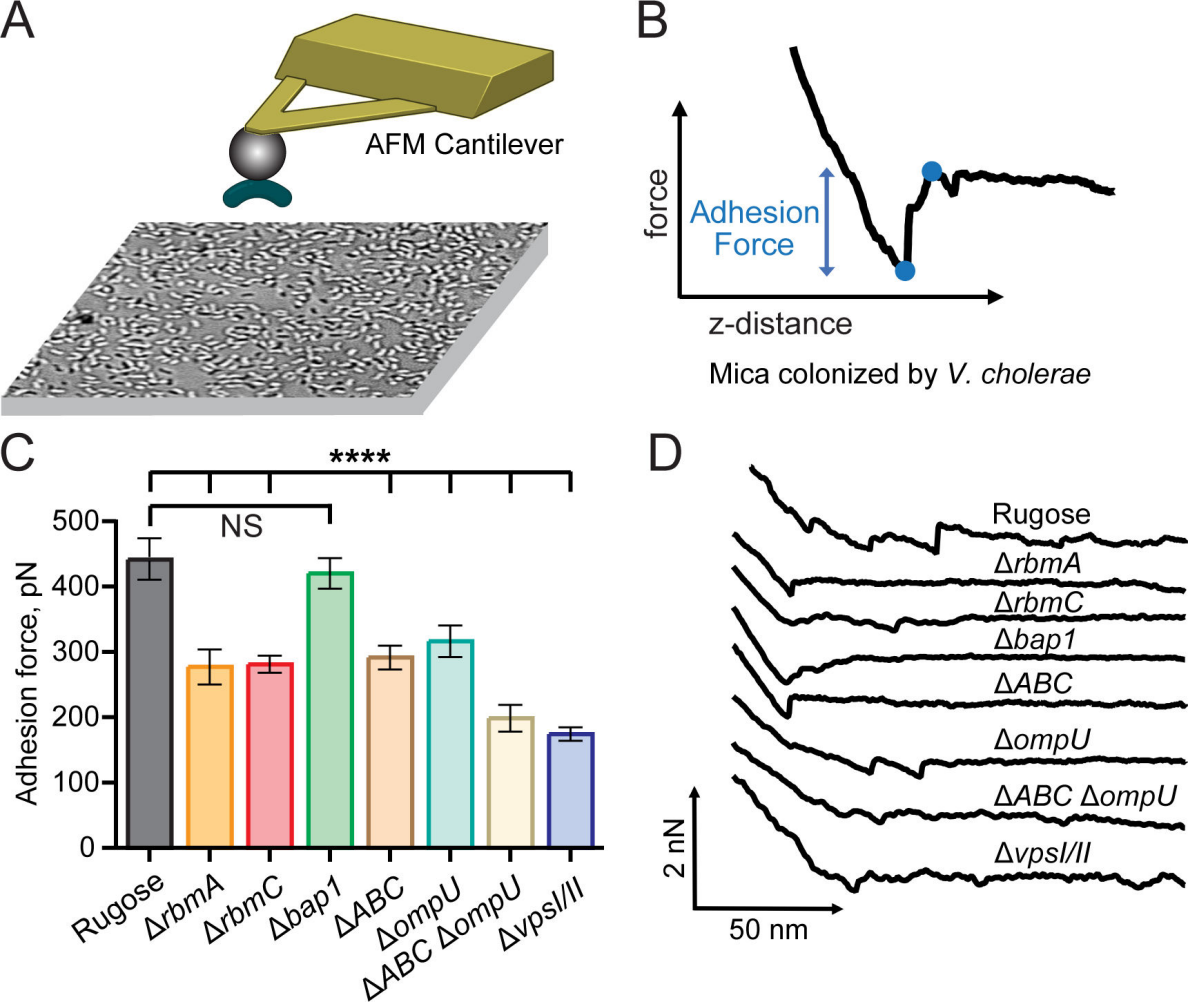
Matrix proteins, OmpU, and VPS contribute to overall adhesion forces of cells in biofilms. (**A**) Cartoon representation of the single cell force spectroscopy experiment. *V. cholerae* cells were attached to a 5 µm bead to obtain surface adhesion measurements from force-distance curves. The surface was colonized with *V. cholerae* cells prior to measurements resulting in mixed cell-cell and cell-surface contributions to the measured adhesion forces. (**B**) Example force-distance curve. The blue points on the curve indicate the minimum and maximum force at the adhesion event. The difference in force between these points is the adhesion force. (**C**) Average adhesion force for the indicated *V. cholerae* strains. Error bars represent standard error of the mean. Asterisks indicate the results of student’s *t*-test relative to the rugose parent strain (****P < 0.00001, NS, not significant). (**D**) Representative force curves for each of the indicated *V. cholerae* strains.

To quantify the role of individual matrix components in biofilm adhesion, we performed a series of single-cell force spectroscopy experiments using a method we have previously used to measure the adhesion of cells and bacterial proteins (48–50). We immobilized individual *V. cholerae* cells on a bead at the end of an atomic force microscopy (AFM) probe and measured the adhesion forces required for a bacterium to attach to a surface (Fig. 5A and B). Previous studies have established that this approach maintains bacterial viability, allowing direct measurement of the adhesion forces between the bacterium and surfaces (51). We chose mica surface because it mimics the negative surface charge of bacterial cells and measured a mixture of cell-cell and cell-surface adhesion forces on an abiotic mica surface (Fig. 5A and B) (52). To determine the contribution of individual proteins to bacterial adhesion, we chose the following strains: Δ*ompU*, Δ*ompT* Δ*rbmA*, Δ*rbmC*, Δ*bap1*, ΔAB (Δ*rbmA*Δ*bap1*), ΔAC (Δ*rbmA*Δ*rbmC*), ΔBC (Δ*bap1*Δ*rbmC*), ΔABC (Δ*rbmA*Δ*bap1*Δ*rbmC*), ΔABCΔ*ompU,* and Δ*vpsI*Δ*vpsII*. Cells of rugose parental strain demonstrated an adhesion force 445 pN. The measured adhesion forces of the Δ*rbmA*, Δ*rbmC,* and ΔABC strains were significantly lower with forces of 281, 285, and 295 pN, respectively. In contrast, the adhesion of the Δ*bap1* strain was similar to the parent strain. While adhesion forces of the ΔAB and ΔBC were similar to the Δ*rbmA* and Δ*rbmC*, adhesion force value for the ΔAC was further decreased to 187 pN (Fig. 5C and D; Fig. S3). Both Δ*ompU* and Δ*ompT* exhibited a decrease in adhesion forces to 320 pN and 259 pN, respectively. In ΔABCΔ*ompU* strain, the adhesion force decreased further to 202 pN. Finally, Δ*vpsI*Δ*vpsII* strain showed the lowest adhesion force values of 178 pN (Fig. 5C and D; Fig. S4).

Collectively our studies show that, under the conditions tested, VPS, both RbmA and RbmC but not Bap1 provide necessary contributions to the adhesion phenotype and that RbmA and RbmC act additively. Bulk adhesion measurements for Bap1 and RbmC, which showed that Bap1 and RbmC contribute to surface adhesion in a surface-dependent manner, are largely consistent with our findings (31). Decreased adhesion measured for the Δ*ompU* strain, which increases VPS production (Fig. 4), and does not alter matrix protein expression (Fig. 3A and B) was unexpected, but consistent with prior reports, which suggest that OmpU may function as an adhesin (53). To further analyze the decreased adhesion phenotype of the Δ*ompU* strain, we compared the hydrophobicity of rugose and the Δ*ompU* strains using the microbial adhesion to hydrocarbon (MATH) assay (54). No significant difference in hydrophobicity was observed between the rugose and Δ*ompU* strains, suggesting that the reduced adhesion force observed in the Δ*ompU* strain cells cannot be attributed to an alteration in hydrophobicity (Fig. S5).

### OMVs carry VPS and alter aggregation within *V. cholerae* biofilm

**Fig 6:**
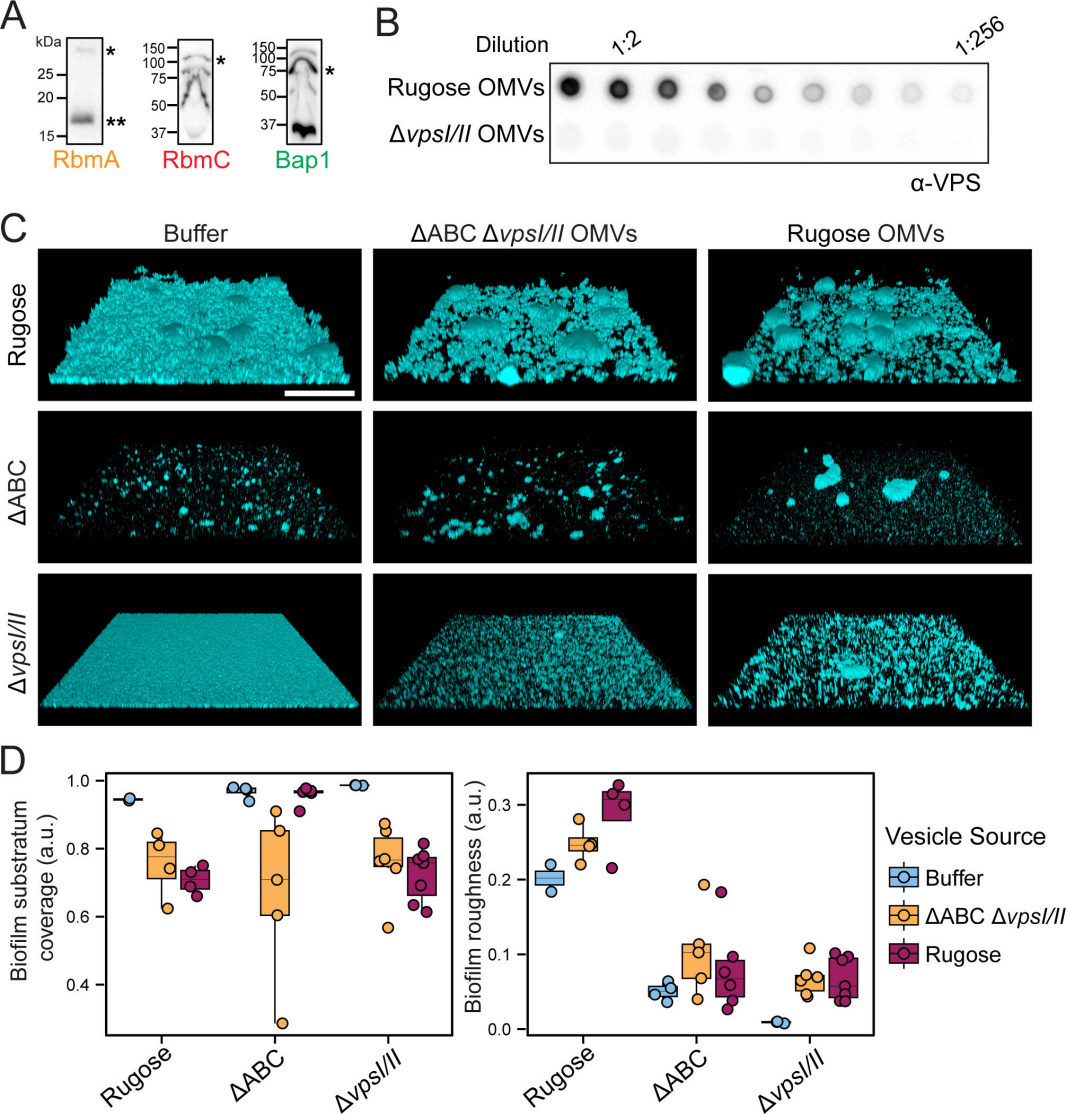
OMVs carry VPS and alter aggregation phenotypes within *V. cholerae* biofilms. (**A**) Immunoblot analysis of RbmA, RbmC, and Bap1 shows their presence on OMVs isolated from biofilm grown cells. Single asterisk indicates full-length protein, while double asterisk indicates proteolytically cleaved protein version. Protein ladder sizes are indicated on the left side of each blot. (**B**) Representative VPS dot-blot shows serial dilutions of OMV samples obtained from biofilms of indicated strains. Serial dilutions of isolated OMVs were applied directly on the membrane that was further probed against VPS-specific antibodies. (**C**) Representative three-dimensional images of the static biofilms formed after 6 h growth at 30°C by strains indicated on the left upon addition of the buffer (first column), Δ*rbmA rbmC bap1* (ABC) Δ*vpsI/II* OMVs (middle column) and rugose OMVs (right column). Scale bars = 100 µm. (**D**) Boxplot representation of the biofilm parameters of the images shown in Fig. 6.C analyzed using BiofilmQ software.

Matrix proteome analysis revealed that OMVs are a significant component of biofilms and that RbmA, Bap1, and RbmC are associated with OMVs. Using an immunoblot analysis, we confirmed the presence of the matrix proteins on OMVs (Fig. 6A). These matrix proteins interact with VPS, and VPS associates with the cell-surface through unknown mechanisms (24, 28, 29, 31). We, therefore, sought to determine if VPS is a part of the OMV cargo and performed immunoblot analysis using isolated OMVs. Vesicles derived from the rugose *V. cholerae* biofilm matrix contained VPS while those isolated from the Δ*vpsI*Δ*vpsII* strain had no VPS associated (Fig. 6B).

Since all essential components of biofilms are associated with OMVs, we hypothesized that OMVs have a direct role in biofilm matrix assembly. To test this idea, we grew the biofilms of rugose, ΔABC (strain lacking matrix proteins), and Δ*vpsI*Δ*vpsII* strains under static conditions and applied OMVs produced by either the rugose strain or by the ΔABCΔ*vpsI*Δ*vpsII* mutant, which lacks VPS and matrix proteins (Fig. 6C). The buffer used to deliver the vesicles was used as a control. We quantified changes to biofilm architecture using a set of parameters via BiofilmQ software. The addition of both OMV types increased cell-aggregate formation and decreased biofilm surface coverage in the rugose and Δ*vpsI*Δ*vpsII* strains compared to the buffer control, indicating a role for OMVs in biofilm matrix assembly. Biofilm roughness, which reflects biofilm density per surface area, increased for the rugose and Δ*vpsI*Δ*vpsII* strain biofilms when mutant OMVs were added and was the highest with OMVs from the rugose strain. The roughness of the ΔABC mutant showed a slight increase irrespective of the vesicle type added (Fig. 6D).

eDNA is an additional constituent of the biofilm matrix of *V. cholerae* (55). As OMVs can transport nucleic acids, we investigated the existence of DNA within *V. cholerae* OMVs and the possible role of DNA associated with OMVs in the formation of biofilms (33). We found that *V. cholerae* OMVs isolated from biofilm-grown rugose cells carry DNA. We then treated the OMVs with DNase and compared how they affected the biofilm properties when added to rugose, ΔABC and Δ*vpsI*Δ*vpsII* biofilms. DNase treatment did not change the effects of the OMVs on the biofilm properties (biofilm roughness and surface coverage) of the rugose and ΔABC strains, but it greatly reduced the OMV-mediated increase in aggregate formation in the Δ*vpsI*Δ*vpsII* (Fig. S6A through C), suggesting that OMV-associated eDNA may interact with VPS to alter biofilm properties. Collectively, these findings indicate that OMVs have the capability to alter biofilm structural properties and suggest that they participate in biofilm matrix assembly.

## DISCUSSION

**Fig 7:**
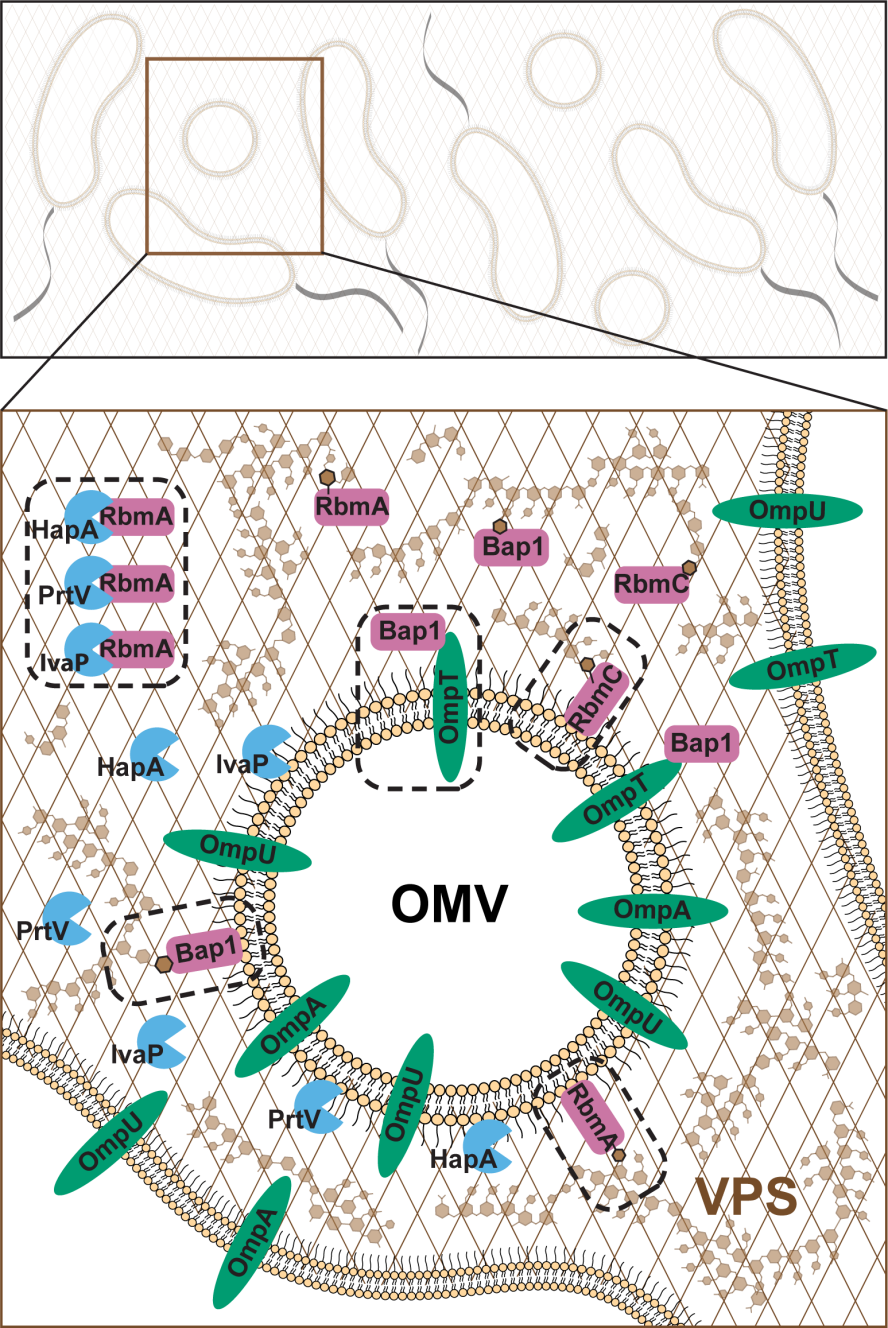
Model of the role of OMVs in biofilm architecture. Biofilm formation and maintenance depend on producing extracellular matrix components—polysaccharides, proteins, nucleic acids, and other biomolecules. Matrix components are involved in cell-cell and cell-matrix interactions and attachment to surfaces. A major component of the *V. cholerae* biofilm matrix is VPS, which is present throughout mature biofilms. VPS can interact with the biofilm matrix proteins RbmA, Bap1, and RbmC; these matrix proteins and VPS are OMV cargos. The proteases IvaP, PrtV, and HapA can all break down RbmA; these proteases are also OMV cargos. Thus, OMVs are likely to play a role in biofilm matrix assembly and matrix remodeling. Matrix proteins are shown in pink, extracellular proteases in blue, and OMPs in green. Dashed rectangles represent confirmed interactions. Figure is modified from Fig. 2 (4).

We defined the proteome of the *V. cholerae* biofilm matrix and found that it is enriched in extracellular, outer membrane, and periplasmic proteins. Our results are consistent with studies that showed the presence of canonical matrix proteins (RbmA, Bap1, RbmC), extracellular proteases, and cell-surface structures in the matrix proteome (27). We found that OMPs and OMVs are abundant in the *V. cholerae* biofilm matrix and showed that matrix proteins, VPS and OmpU, contribute to cell-to-cell and cell-to-surface adhesion forces in *V. cholerae*. We determined that the primary OMP OmpU influences biofilm formation by regulating VPS production and that OMVs contribute to biofilm matrix assembly by harboring VPS and matrix proteins (Fig. 7).

OMPs serve various functions in Gram-negative bacterial physiology, environmental adaptation, and virulence. The significance of OMPs in biofilm formation, structure, and function is emerging. OmpU is well-conserved across *Vibrio* species and is the most abundant and best-studied OMP in *V. cholerae* (56). OmpU confers resistance to host-derived antimicrobial peptides, and bile connects envelope stress to activation of the σE pathway, facilitates the adhesion of *V. cholerae* to mammalian cells, and modulates host immune responses (45, 57–59). We found that OmpU regulates biofilm matrix production, expanding the role OmpU plays in *V. cholerae* cellular processes. The virulence regulator ToxR positively regulates *ompU* expression, and we found that both Δ*ompU* and Δ*toxR* strains form hyper-corrugated colonies and the Δ*toxR* phenotype was solely due to decreased OmpU accumulation. These results suggest that ToxR regulates biofilm formation through regulation of OmpU.

Strains lacking OmpT and OmpA had unaltered ability to form biofilms. Expression of *ompA* and *ompT* together with *rbmC* encoding one of the key matrix proteins is under control of the small RNA VrrA. Under stress conditions, actively expressed VrrA suppresses the production of OmpA and OmpT which minimize membrane conductivity and increase vesiculation and simultaneously represses the expression of RbmC to eventually promote biofilm detachment (36, 60, 61). Thus, the contributions of OmpT and OmpA to biofilm formation could be conditional and governed by environmental stress responses. In *P. aeruginosa*, the major OMP, OprF, is an abundant matrix protein and promotes or represses biofilm formation depending upon growth conditions (62, 63). OprF interacts with surface-exposed lectin LecB, which binds *P. aeruginosa* exopolysaccharide Psl, thereby promoting biofilm matrix assembly and stability through increased retention of cells and exopolysaccharides in biofilms (64). In *E. coli*, OmpA, the homolog of OprF, represses or enhances biofilm formation depending on the substratum properties (65, 66). Although its presence in the biofilm matrix was not experimentally confirmed, OmpA of *Acinetobacter baumannii* helps robust biofilm formation on abiotic surfaces (67). *E. coli* biofilms utilize cellulose as the main polysaccharide, and OmpA in *E. coli* enhances biofilm formation on polystyrene surfaces by down-regulating cellulose production (65).

OMVs are derived from the outer membrane of the bacteria and contain outer membrane proteins, lipids, and periplasmic contents (32, 33). *V. cholerae* releases OMVs as part of its virulence and defense mechanisms. We found that OMVs are a component of the *V. cholerae* biofilm matrix and determined the proteome of OMVs isolated from the matrix. We found that the cargo of OMVs isolated from biofilm-grown cells is similar to the composition of OMVs produced by planktonic cells, demonstrating the ability of *V. cholerae* OMVs to carry matrix proteins, extracellular proteases, protein components of cell-surface structures, OMPs, and several periplasmic proteins (27, 42). We analyzed the role OMVs play in biofilm formation using complementary approaches. Immunoblot analysis confirmed that OMV’s cargo includes key matrix proteins RbmA, RbmC, Bap1, and VPS. These matrix proteins can directly interact with VPS (29, 31). We showed that protein, VPS, and DNA cargo on OMVs impact biofilm architecture. The ability of a strain deficient in matrix proteins or VPS production was altered upon the addition of isolated OMVs from the parent strain. Importantly, vesicles prepared from the strain unable to produce RbmA/RbmC/Bap1/VPS still affected microcolony formation within the biofilm although not in the same way as OMVs isolated from the parent strain. We further found that OMV-associated DNA cargo impacts biofilm properties likely through interaction with VPS. Taken together, our data suggest a role for OMVs in biofilm formation. Furthermore, this role is likely not defined simply by the ability of OMVs to carry the matrix proteins and VPS, suggesting that other OMV cargo also plays a role in biofilm matrix assembly.

In *V. cholerae*, one mechanism of OMV formation is through the VacJ/Yrb ABC transport system involved in maintaining lipid asymmetry in the OM; disruptions in the Yrb system increase OMV production (37). Another OMV production mechanism involves decreasing the production of OmpA, which links OM to the peptidoglycan. OmpA levels are negatively correlated with the number of OMVs, and sRNA VrrA increased OMV production comparable to OmpA depletion (36). In the rugose genetic background, the deletion of *ompA* did not alter colony biofilm properties. Another player in OMV production is DegP. This protein functions as a protease and chaperone that serves as a protein quality control factor in the bacterial envelope and plays a crucial role in determining the composition of OMVs such as those involved in biofilm matrix formation and various substrates of type II secretion (42). A DegP mutant in the EC956 *V. cholerae* strain showed a biofilm defect (42). We deleted *degP* in the rugose genetic background and observed no changes in colony biofilm properties (Fig. S7).

The biofilm matrix protein Bap1 plays a role in OMV-mediated antimicrobial peptide resistance in *V. cholerae* El Tor O1 (68). Studies on the role of *V. cholerae* O1 El Tor A1552 OMVs in antimicrobial peptide (AMP) resistance specifically to Polymyxin B and LL-37 showed that OMV protein profiles from *V. cholerae* cultures grown in the presence of Polymyxin B are different from cultures grown without AMPs. When bacteria are exposed to Polymyxin B, they release larger OMVs that contain biofilm matrix protein Bap1. Bap1 binds to the OmpT porin on OMVs by means of the LDV domain of OmpT. Moreover, OMVs from *V. cholerae* cultures grown with Polymyxin B are more protective against LL-37 than OMVs from *V. cholerae* cultures grown without AMPs or in the presence of LL-37. This cross-resistance is caused by Bap1 found in OMVs of *V. cholerae* grown with Polymyxin B (68). Collectively, these studies suggest that *in vivo* biofilm formation and OMV production in biofilms provide AMP resistance during *V. cholerae* infection. These observations suggest that OMVs produced by different mechanisms impact biofilm formation differently; whether this is due to the type or abundance of OMV cargo remains to be determined.

Analysis of the biofilm matrix in diverse organisms makes it increasingly clear that OMVs are core components. In *P. aeruginosa* biofilms, OMVs are predominant, and most biofilm matrix proteins are derived from OMVs. Moreover, the protein content of planktonic and biofilm OMVs is distinct, which may reflect the contrasting physiological states of planktonic and sessile cells (69–71). OMVs produced by *P. putida* and *H. pylori* enhance biofilm formation (72, 73). OMVs are also present in the *Vibrio fischeri* biofilm matrix, and OMV production is increased in biofilm-overproducing cells depending on the symbiosis polysaccharide (*syp*) locus (74). Furthermore, *V. fischeri* changes the composition of its OMVs when it transitions from its environmental reservoir to host tissues (75). In contrast, vesicles produced by the plant pathogen *Xylella fastidiosa* prevent cellular attachment to environmental and host surfaces, restrict biofilm formation, and promote an “exploratory” lifestyle (76). While it is becoming increasingly evident that OMPs and OMVs contribute to the assembly and structure of the biofilm matrix, molecular mechanisms of OMV production, where and when OMVs are produced during biofilm formation, are yet to be determined. OMV outer membrane proteins can interact with polysaccharides and proteins, thereby promoting their assembly and organization. Thus, OMVs can function as nucleation sites for matrix assembly and help stabilize biofilm structure. OMVs can also contribute to emergent biofilm properties such as nutrient acquisition through degradative enzymes and genetic exchange. Understanding the function of OMVs in biofilm biology can aid in developing strategies to combat biofilm-related infections and disrupt biofilm formation. Our findings contribute to the growing body of evidence indicating that OMVs play a significant role in diverse cellular processes during the infection cycle of *V. cholerae*.

## MATERIALS AND METHODS

### Bacterial strains and culture conditions

All bacterial strains and plasmids used in this study are listed in Table S4. *E. coli* CC118 λ pir and DH5α λ pir were used for DNA and plasmid construction and manipulation. *E. coli* S17 λ pir was used for conjugation with *V. cholerae. E. coli* and *V. cholerae* strains were grown aerobically in Luria-Bertani (LB) broth (1% tryptone, 0.5% yeast extract, 1% NaCl, pH 7.5) at 37°C and 30°C, respectively, with 200 rpm shaking. Granulated agar (BD Difco, Franklin Lakes, NJ) was added at 1.5% (wt/vol) for LB agar medium. Medium additives, when necessary, were used at the following concentrations: 100 µg/mL rifampicin (Rif), 100 µg/mL ampicillin (Amp), and 15 µg/mL gentamicin (Gm). All overnight *V. cholerae* liquid cultures were obtained by inoculating five single colonies from LB-agar plates into 5 mL LB media.

### Strain and plasmid construction

Plasmids were constructed using standard cloning methods or the Gibson Assembly recombinant DNA technique (New England BioLabs, Ipswich, MA). Restriction enzymes were purchased from New England Biolabs.

Gene in-frame deletions and chromosomal protein tagging were performed using an allelic exchange of the native open reading frame (ORF) with the truncated or tagged ORF, as previously described (77). Polymerase chain reactions (PCRs) were carried out with primers purchased from Integrated DNA Technologies and Q5 High-Fidelity master mix (New England BioLabs). Sequencing of plasmid constructs was performed at the Genewiz/Azenta Life Sciences (Chelmsford, MA) company. All *V. cholerae* sequences were amplified from genomic DNA isolated from *V. cholerae* A1552 strain.

Both fluorescent protein tagged and chromosomal complementation strains were generated through a Tn7-based system that inserts in the genomic region between loci VC0487 and VC0488, as described previously (78). Triparental conjugation was performed, with donor *E. coli* S17-1 λ *pir* cells carrying either pGP704::Tn7-GFP (or desired promoter-gene in place of GFP), as well as a second helper *E. coli* S17-1 λ *pir* harboring pUX-BF13 carrying the Tn7 transposase gene. Transconjugants were selected on thiosulfate-citrate-bile salts-sucrose (Difco) agar medium containing 15 µg/mL gentamicin at 30°C. Tn7 insertion strains were verified by PCR analysis.

For the *ompU* overexpression construct, the open reading frame of *ompU* was cloned into pMMB67EH, which contains the IPTG inducible *Ptac* system. Resulting plasmid was transformed into the *E. coli* S17-1 λ *pir* donor strain and introduced into the *V. cholerae* A1552 strain by conjugation.

### Isolation and quantification of extracellular matrix

Cultures grown overnight at 30°C with 200 rpm shaking were diluted to OD_600_ 0.1 and 200 µL were spread on 20 mL LB 100 × 15 mm LB agar plates (1 plate per time point per biological replicate), overlaid with sterile dialysis membrane (Fisher Scientific, Waltham, MA). Samples were grown at 30°C for 24 h before biofilm was harvested and resuspended in 1× phosphate-buffered saline (PBS, pH 7.4) containing SigmaFast EDTA-free protease inhibitor cocktail (Sigma-Aldrich, St. Louis, MO). Normalization of samples was carried out by adjusting each sample to the same OD_600_. Equal volumes of samples were transferred to new conical tubes and constantly rotated, at 4°C, for 2 h. Crude extracellular matrix was separated from cells and debris by centrifugation twice at 15,000×*g* for 15 min at 4°C. Supernatant was filtered through 0.45 µm membranes using 0.5 mL Ultrafree-MC centrifugal filters (Millipore Sigma, Burlington, MA). Samples were transferred to Eppendorf Safe-Lock Natural tubes (Eppendorf, Germany). Bovine serum albumin solution and sodium deoxycholate were added to each sample to final concentrations of 35 µg/mL and 0.02%, respectively. Samples were vortexed to mix and incubated on ice for 1 h. Trichloroacetic acid was added to each sample to a final concentration of 12.5%. Samples were vortexed to mix and incubated overnight, shaking, at 4°C. Samples were centrifuged at 21,130×*g* for 1 h at 4°C. Supernatant was discarded, and resulting pellets were resuspended/re-submerged in 1.5 mL of ice-cold acetone followed by second centrifugation at 21,130×*g* for 1 h at 4°C. Supernatant was discarded, and resulting pellets were allowed to dry in the fume hood for 10–15 min. Samples were resuspended in 120 µL of 1× PBS/protease inhibitor cocktail mixture. Resuspended samples were boiled at 100°C for 45 min and mixed intermittently, ensuring the entirety of the sample is resuspended.

To prepare isolated biofilm matrix protein samples for subsequent mass-spectrometry (LC-MS/MS) analysis, pellets were resuspended in 400 µL of 5% SDS/protease inhibitor cocktail/50 mM triethylammonium bicarbonate after drying in the fume hood. Resuspended samples were boiled at 100°C for 45 min and mixed intermittently. Protein content was determined by BCA protein assay (Fisher Scientific), and 100–150 µg aliquots were flash frozen in liquid nitrogen. Samples were analyzed at the UC Davis Proteomics Core Facility.

Matrix protein abundance was calculated using ImageJ-Fiji software based on the measured band intensity on performed immunoblots. Intensities of bands corresponding to RbmA, RbmC, and Bap1 were normalized by measured intensities of corresponding BSA bands (BSA was used as loading control), and then protein abundance in the *ompU* mutant was calculated relative to that in rugose strain.

### Outer membrane vesicle isolation

Cultures grown overnight at 30°C with 200 rpm shaking were diluted to OD_600_ 0.1 and 500 µL were spread on 50 mL 150 × 15 mm LB agar medium plates, overlaid with sterile dialysis membrane (Fisher Scientific) (10 plates per strain per biological replicate were used). Samples were grown at 30°C for 24 h before biofilm was harvested and resuspended in 1× PBS (pH 7.4) supplied with SigmaFast EDTA-free protease inhibitor cocktail (Sigma-Aldrich). Normalization of samples was carried out by adjusting each sample to the same OD_600_. Equal volumes of samples were transferred to new conical tubes and rotated overnight at 4°C. Crude extracellular matrix was separated from cells and debris by centrifugation twice at 13,000×*g* for 10 min at 4°C. Supernatant was filtered through 0.45 µm membranes using 50 mL tube top vacuum filter system (Corning, Corning, NY). Crude OMVs were pelleted by centrifugation at 150,000×*g* for 3 h at 4°C. Pellets were submerged in rehydrated 120 µL of 50 mM HEPES buffer (pH 6.8) overnight at 4°C and then resuspended. Resulted samples were used for the immunoblot analysis and biofilm experiments.

To prepare isolated OMV samples for subsequent mass spectrometry (LC-MS/MS) analysis, OMV samples were further adjusted to 45% Optiprep and fractionated using an Optiprep density gradient of the following percentages: 10%, 15%, 20%, 25%, 30%, and 35%. Samples were fractionated at 292,700×*g* for 3 h at 4°C. Aliquots of equal volume were removed sequentially from the gradient and analyzed via SYPRO protein staining and immunoblot using antibodies against OmpU and FlaA. The fractions enriched with OmpU, hence OMVs, were pooled and separated from Optiprep via centrifugation at 200,000×*g* for 3 h at 4°C. OMV pellets were resuspended in DPBS (10 mM Na_2_HPO_4_, 1.8 mM KH_2_PO_4_, 137 mM NaCl, 2.7 mM KCl), and resulting samples were analyzed at the UC Davis Proteomics Core Facility.

### Identification of the total matrix and matrix-associated vesicles proteomes

#### Tryptic digestion of the matrix and OMVs proteomic samples

Tryptic digestion was carried out via suspension-trap (S-Trap) devices (ProtiFi, Fairport, NY). Disulfide bonds were reduced with dithiothreitol and alkylated with iodoacetamide in 50 mM TEAB. Then, the enzymatic digestion: a first addition of trypsin 1:100 enzyme: protein (wt/wt) for 4 h at 37°C, followed by a boost addition of trypsin using the same wt/wt ratios for overnight digestion at 37°C. Peptides were eluted from S-Trap by sequential elution buffers of 100 mM TEAB, 0.5% formic acid, and 50% acetonitrile 0.1% formic acid. The eluted tryptic peptides were dried in a vacuum centrifuge and re-constituted in 0.1% trifluoroacetic acid. These were subjected to fluorescent peptide (FP) assay for concentration evaluation. Based on the FP, 0.6 µg of total peptide for each sample was subjected to LC-MS/MS analysis.

#### LC-MS/MS

Performed on an ultra-high pressure nano-flow Easy nLC (Bruker Daltonics, Fremont, CA). Liquid chromatography was performed at 40°C and with a constant flow of 400 nL/min on a PepSep 150 µm × 25 cm C18 column (PepSep, Denmark) with 1.5 µm particle size (100 Å pores) and a ZDV captivespray emitter (Bruker Daltronics). Mobile phases A and B were water with 0.1% formic acid (vol/vol) and 80%/20%/0.1% ACN/water/formic acid (vol/vol/vol), respectively. Peptides were separated using a 120 min gradient. Eluting peptides were further separated using TIMS (trapped ion mobility spectrometry) on a Bruker timsTOF Pro 2 mass spectrometer. Mass spectrometry data were acquired using the dda PASEF method (79). The acquisition scheme used was 100 ms accumulation, 100 ms PASEF ramp (at 100% duty cycle) with up to 10 PASEF MS/MS scans per topN acquisition cycle. The capillary voltage was set to 1,700 V, and the capillary gas temperature was set to 200°C. The target value was set at 20,000 a.u. with the intensity threshold set at 500 a.u. The *m*/*z* range surveyed was between 100 and 1,700. Precursor ions for PASEF-MS/MS were selected in real time from a TIMS-MS survey scan using a non-linear PASEF scheduling algorithm. The polygon filter (200–1,700 *m*/*z*) was designed to cover ions within a specific *m*/*z* and ion mobility plane to select multiply charged peptide features rather than singly charged background ions. The quadrupole isolation width was set to 2 Th for *m*/*z* <700 and 3 Da for *m*/*z* 800.

#### Mass spectrometry data analysis

Mass spectrometry raw files were searched using Fragpipe 19.1 (80) against the UniProt *Vibrio cholerae serotype O1* (strain *ATCC 39315*/*El Tor Inaba* N16961) proteome (UP000000584). Decoy sequences and laboratory contaminants sequences were generated and appended within Fragpipe. Default search settings were used. Carbamidomethylation of cysteine residues was set as a fixed modification, and methionine oxidation and acetylation of protein N termini as variable modifications. Decoy False Discovery Rates were controlled at 1% maximum using the Peptide and Protein prophet algorithms. Label-free protein quantification was performed with the IonQuant algorithm with Match Between runs turned on (default settings) (81).

Search results were loaded into fragpipe-analyst, a variant of LFQ-Analyst (82), for visualization and statistical differential expression purposes. Proteins that contained similar peptides and could not be differentiated based on MS/MS analysis alone were grouped to satisfy the principles of parsimony. Proteins sharing significant peptide evidence were grouped into clusters. Representative heatmaps were generated using the ComplexHeatmap package in R using maxLFQ intensity values (83). The rows in the heatmaps were clustered hierarchically.

### Cryo-Electron tomography

Biofilms of the rugose strain of *V. cholerae* were cultivated as described above. The periphery of a mature biofilm was gently stabbed with a 10 µL pipette tip and briefly resuspended in 1× PBS. Five microliters of sample was then deposited onto freshly glow-discharged holey carbon grids (Quantifoil, Germany, Cu R2/1, 200 mesh). The grids were first back blotted with filter paper to remove the PBS; then we added 5 µL PBS with ≈5% (vol/vol) glycerol for cryo-protectant and incubated for ≈1 min. Afterward, the grids were back-blotted with filter paper for ≈3–5 s before being plunge-frozen in liquid ethane using a homemade plunger apparatus as previously described (84, 85).

Cryo-FIB milling was performed in an Aquilos system (Thermo Fisher Scientific) as follows: under liquid nitrogen conditions, the plunge-frozen grids were clipped into cryo-FIB AutoGrids and mounted into a specimen shuttle. The samples were sputter-coated with Pt (1 kV, 15 mA, 15 s) to improve the overall sample conductivity. To protect the biofilm samples from radiation damage, they were coated by an organometallic Pt layer (4–5 µm thick) using the gas injection system. Biofilms (several µm in thickness) were milled into a thin ≈150–200 nm lamella by a gallium ion beam at 30 kV with a stage tilt angle of around 17°. The ion beam current was sequentially reduced as the lamella became thinner during the milling process: 0.5 nA for *t* ≥ 3 µm, 0.3 nA for *t* ≥ 1 µm, 0.1 nA for *t* ≥ 500 nm, 0.05/0.03 nA for final polishing. The lamella was finally sputter-coated with a thin Pt layer (1 kV, 10 mA, 5 s) to prevent potential charging issues during cryo-ET imaging.

The lamellae were visualized in a 300 kV Titan Krios electron microscope (Thermo Fisher Scientific) equipped with a K3 direct electron detector and energy filter (Gatan). SerialEM (86) was used to collect single-axis tilt series with a cumulative dose of ~90 e-/Å covering angles from −51° to 51° (3° tilt step). Images were acquired at 26,000× magnification, resulting in an effective pixel size of 3.384 Å at the specimen level. All recorded images were first drift corrected by the software MotionCor2 (87) and then stacked by the software package IMOD (88). IMOD was used to align the tilt series and reconstruct tomograms. IMOD was then used to visualize the OMVs in the resulting tomograms in order to estimate the number and volumes of the OMVs.

### Scanning electron microscopy

Cultures grown overnight at 30°C with 200 rpm shaking were diluted to OD_600_ 0.1 and 100 µL were spread onto 20 mL LB agar plates overlaid with a sterile dialysis membrane (Fisher Brand, Fisher Scientific), using a sterile cell spreader. Samples were grown over 24 h at 30°C. Sterile forceps were used to place sterile 12 mm diameter coverslips (Chemglass, Vineland, NJ) on the bacterial lawn. Slides were gently pressed onto the sample; then slides were lifted and placed into a dish containing 2.5% glutaraldehyde for 1 h at room temperature. Samples were dehydrated by transferring sequentially into dishes containing 30%, 50%, 70%, and 90% ethanol. Samples were stored in 100% ethanol at room temperature until imaging. The samples were critical point dried, sputtered with ~20 nm of gold, and imaged in an FEI Quanta 3D Dualbeam SEM operating at 5 kV and 6.7 pA.

### Analysis of spot, colony, and pellicle morphology

For morphology imaging, 20 mL of LB agar was added per plate, and the plates were dried at room temperature for 48 h before use. The overnight cultures were diluted 1:200 for spot morphology analysis, and 3 µL was spotted on a plate with three spots per plate total. The overnight cultures were diluted to 10^−9^ for colony morphology, and spots and colonies were grown at 30°C for 72 h. For pellicle analysis, the overnight cultures were diluted 1:200, and 200 µL of resulting dilutions was placed in a clear 96-well plate (Thermo Fisher Scientific) and grown statically at 30°C for 24 h. Spot, colony, and pellicle morphology were imaged with the Zeiss Stemi 2000-C microscope equipped with Zeiss AxioCam ERc 5 s Microscope Camera. Morphology experiments were carried out with a minimum of two biological replicates.

### Immunoblot analysis

For analysis of protein abundance, equal volumes of resulting OMVs and biofilm matrix protein preparations were combined with 5× Laemmli sample buffer and loaded onto 10- or 15-well 12% SDS-PAGE. After SDS-PAGE electrophoresis, the proteins were transferred onto a PVDF membrane (Immobilon, 0.45 µm, Millipore Sigma) and probed via immunoblotting (90 and 60 min with primary and secondary antibodies, respectively). The following antibodies were used for immunoblotting: anti-RbmA (1:1,000) (28), anti-RbmC (Cocalico; 1:1,000), anti-Bap1 (Cocalico; 1:1,000), anti-OmpU (GenScript; 0.5 µg/mL), and mouse anti-rabbit horseradish peroxidase-conjugated (Promega, 1:2,500). Membranes containing isolated biofilm matrix proteins were stripped and additionally probed for BSA as loading and processing control using polyclonal anti-BSA (Invitrogen; 1:1,000) and mouse anti-rabbit horseradish peroxidase-conjugated (Promega, 1:2,500) antibodies. The immunoblots were developed with the SuperSignal West Pico chemiluminescent kit (Pierce, Fisher Scientific). Immunoblot analyses were carried out with a minimum of two biological replicates.

### VPS isolation and quantification

VPS purification was adapted from a previously published protocol (24). Cultures grown overnight at 30°C with 200 rpm shaking were diluted to OD_600_ 0.1 and 100 µL were spread onto 20 mL LB agar plates. Samples were grown over 24 h at 30°C. Upon harvesting, the cells were resuspended in 10 mM Tris (pH 8.0) and adjusted to the same cell density. Equal volumes of cell suspensions were pipetted into 2 mL Eppendorf tubes and rotated for 1 h at 4°C. To separate the matrix material from the cells, the suspension was centrifuged twice at 15,000×*g* for 30 min at 4°C. The supernatant fraction was recovered each time and, after the second centrifugation step, precipitated in 3 volumes of ice-cold ethanol at −20°C overnight. Crude VPS was then pelleted at 21,130× *g* for 30 min at 4°C followed by a 70% ethanol wash. The VPS pellets were then dried on ice and resuspended in 500 µL nuclease buffer (40 mM Tris/HCl pH 8.0, 10 mM MgCl_2_, 2 mM CaCl_2_, 0.05% NaN_3_) incubated with DNase I and RNase A at final concentrations of 2 units/mL and 0.25 units/mL, respectively. The resulting samples were incubated at 37°C, shaking at 200 rpm for 3 h, and Proteinase K was added at a final concentration of 250 µg/mL followed by further overnight incubation at 37°C, shaking at 200 rpm. 1:1 Phenol/chloroform extractions were carried out in duplicate, followed by overnight precipitation with 3× volumes of 100% ethanol at −20°C. VPS was pelleted twice by centrifugation at 15,000×*g* for 30 min at 4°C, intermittently resuspended with 70% ethanol. Pellets were then air-dried and resuspended in 100 µL sterile Millipore water.

For quantification, the VPS was serially diluted, and 3 µL of each dilution was spotted on nitrocellulose membrane (Amersham Protran, 0.2 µm), followed by immunoblot analysis with anti-VPS antiserum (1:1,000) and goat anti-rabbit horseradish peroxidase-conjugated antibody (1:2,500). The immunoblots were developed with the SuperSignal West Pico chemiluminescent kit (Pierce) and quantified using Fiji imageJ software. VPS immunoblot analyses were carried out with three biological and two technical replicates.

### Identification of the whole cell proteome of the Rugose and Δ*ompU* strains

#### Whole-cell proteomics sample preparation and Tandem Mass Tag (TMT) labeling

Whole-cell proteomes were obtained from colony biofilms. Cultures grown overnight at 30°C with 200 rpm shaking were diluted 1:200, and 3 µL of each culture was spotted on a 20 mL LB-agar plate. Plates were incubated for 48 h at 30°C, and three biological replicates were used for further sample preparation. EasyPep Mini MS Sample Prep Kit and TMTpro 16plex Label Reagent Set (both Thermo Fisher Scientific) were used for complete sample preparation, and manufacturer recommendations were followed. In brief, *V. cholerae* colony biofilm was resuspended in 100 µL of Lysis solution containing 1 µL of Universal Nuclease. The resulting cell suspensions were sonicated for 2 min (10 s on/20 s off, 20% amplitude) and centrifuged at 21,130×*g* for 30 min at 4°C. The protein concentration of the supernatants was determined using BCA protein assay (Thermo Fisher Scientific), and ≈30–50 µg of protein sample was transferred into a new microcentrifuge tube. Sample reduction, alkylation, and protein digestion were performed as the manufacturer’s protocol suggested.

TMT labeling was performed after protein digestion and before peptide clean-up following the manufacturer’s protocol. In brief, each TMT reagent vial (0.5 mg) was dissolved in 20 µL of anhydrous acetonitrile (LC-MS grade, Thermo Fisher Scientific), and 10 µL of the mixture was added to each sample. The reaction was allowed to proceed for 60 min at room temperature and then quenched and acidified for 15 min by adding 50 µL of 5% hydroxylamine/20% formic acid. Sample pH was verified (pH ˂ 4), and peptide clean-up was performed following the manufacturer’s protocol. Digested samples were dried in a vacuum centrifuge and analyzed by the UC Davis Proteomics Core Facility.

#### Fractionation

TMT-labeled samples were reconstituted in 0.1% TFA and the pH is adjusted to 2 with 10%. The combined sample (20 µg) was separated into eight fractions by Pierce High pH Reverse-Phase peptide Fractionation kit (Thermo Scientific) with an extra wash before separation to remove extra labels. The eight fractions were dried almost to completion.

#### LC-MS/MS

LC separation was done on a Dionex nano Ultimate 3000 (Thermo Scientific) with a Thermo Easy-Spray source. The digested peptides were reconstituted in 2% acetonitrile/0.1% trifluoroacetic acid, and 5 µL of each sample was loaded onto a PepMap 100 Å 3U 75 µm × 20 mm reverse phase trap where they were desalted online before being separated on a 100 Å 2U 50 µm × 150 mm PepMap EasySpray reverse phase column. Peptides were eluted using a 120 min gradient of 0.1% formic acid (A) and 80% acetonitrile (B) with a flow rate of 200 nL/min. The separation gradient was run with 0%–3% B over 3 min, 6%–10% B over 3 min, 10%–23% B over 74 min, 23%–50% B over 15 min, 50%–99% B over 5 min, a 4 min hold at 99% B, and finally 99%–2% B held at 2% B for 16 min.

#### MS3 synchronous precursor selection Workflow

Mass spectra were collected on a Fusion Lumos mass spectrometer (Thermo Fisher Scientific) in a data-dependent MS3 synchronous precursor selection (SPS) method. MS1 spectra were acquired in the Orbitrap, 120 K resolution, 50 ms max inject time, 100% normalized AGC target. MS2 spectra were acquired in the linear ion trap with a 0.7 Da isolation window, CID fragmentation energy of 30%, rapid scan speed, 120 ms max inject time, 200% normalized AGC target. MS2 ions were isolated in the iontrap and fragmented with an HCD energy of 45%. MS3 spectra were acquired in the orbitrap with a resolution of 50 K and a scan range of 100–500 Da, 200 ms max inject time, and 200% normalized AGC target.

#### Proteome discoverer Search

Tandem mass spectra were processed and searched using Proteome Discoverer version 2.5. All spectra were recalibrated using the PD 2.5 recalibration node with the default parameters. MS3 reporter ions were quantified and detected using the most confident centroid with a mass tolerance of 20 ppm. MS/MS spectra were searched using SEQUEST-HT with trypsin enzyme specificity; max 2 missed cleavages, fragment ion tolerance of 0.6 Da, and precursor mass tolerance of 10 ppm. SEQUEST HT was set up to search the UniProt *Vibrio cholerae serotype O1* (strain *ATCC 39315*/*El Tor Inaba* N16961) proteome (UP000000584) and common laboratory contaminants from http://thegpm.org.crap. Carbamidomethyl of cysteine, TMT10plex of lysine, and peptide n-termini were fixed modifications. Oxidation of methionine and acetyl of the protein n-terminus were specified in Sequest (XCorr Only) as variable modifications. SEQUEST HT results were processed with a percolator with a maximum Delta Cn of 0.05. Target decoy FDRs were set at 1% with validation based on *q*-value. A report was exported from Proteome Discoverer and imported into SimpliFi (https://simplifi.protifi.com/) for QC and statistical analysis (Protifi, Farmingdale, NY).

### Adhesion force measurement and analysis

AFM probes for single cell force spectroscopy methods were prepared as described by Beaussart et al. (89). We attached silica microspheres (Polysciences, Warrington, PA) of diameter 5.0 µm to tipless AFM cantilevers (PNP-TR-TL-Au, NanoWorld, Switzerland) using UV curable optical adhesive (NOA 61, Norland Products). The probes were immersed in 10 mM tris buffer (pH 8.5) containing 4 mg/mL Dopamine hydrochloride (H8502, Sigma Aldrich) for 1 h, after which the probes were rinsed with Milli-Q and dried with N-2.

*V. cholerae* cells were grown overnight on LB agar at 30°C. Individual colonies were scraped from the plate, transferred to liquid LB broth, and grown overnight at 30°C. The culture was briefly vortexed to break up any biofilm that formed at the air-liquid interface and allowed to settle for 15 min. We deposited 15 µL of planktonic cells on freshly cleaved muscovite mica (71855-01, Electron Microscopy Sciences, Hartfield, PA). The cells were allowed to colonize the surface for at least 1 h. Using a Cypher ES atomic force microscope (Asylum Corporation, Oxford Instruments, Santa Barbara, CA), individual cells were attached to the polydopamine-coated AFM probe. Specifically, the probe was lowered in the cell-containing phosphate-buffered saline (PBS) until stable contact with the mica surface was detected. Contact was held with a force of 5–10 nN for 3 min before withdrawing.

Force spectroscopy was performed at 30°C using an Asylum Cypher ES atomic force microscope. Probe calibration and spring constant determination were achieved via the thermal noise method. Curves were generated using a constant approach and retraction velocity of 1 µm/s. A retraction was triggered upon applying a 1 nN force following a 3 s dwell on the surface. For each cell attached to the probe, we acquired around 20 force curves.

Experiments were performed on the same mica used to attach the cells to the AFM probe. Therefore, the surface is coated in *V. cholerae* cells, and the adhesion forces we have measured are a combination of cell-cell and cell-surface adhesion.

Each force curve was analyzed individually in Igor Pro software. The adhesion force was taken to be the difference between the minimum and baseline forces in the retraction curve. Force curves that did not show a clear adhesion event were excluded from the analysis. The following amount of force curves was obtained per strain indicated: 259 force curves across 13 cells for the Rugose strain, 60 force curves across 4 cells for the Δ*rbmA* strain, 242 force curves across 11 cells for the Δ*bap1* strain, 274 force curves across 14 cells for the Δ*rbmC* strain, 21 force curves across 2 cells for the Δ*rbmA*Δ*rbmC* strain, 59 force curves across 4 cells for the Δ*rbmA* Δ*bap1* strain, 51 force curves across 4 cells for the Δ*rbmC*Δ*bap1* strain, 183 force curves across 8 cells for the ΔABC strain, 96 force curves across 6 cells for the Δ*ompU* strain, 140 force curves across 7 cells for the Δ*ompT* strain, 34 force curves across 3 cells for the ΔABC Δ*ompU* strain, and 236 force curves across 15 cells for the Δ*vpsIΔvpsII* strain.

### Biofilm assay and confocal laser scanning microscopy

OMVs were isolated and resuspended in a 50 mM HEPES buffer (pH 6.8), and protein content was determined by BCA protein assay (Thermo Fisher Scientific). One hundred fifty to three hundred micrograms of OMV protein was applied in the uncoated eight well µ-Slide with polymer coverslip bottom (Ibidi, Germany) and allowed to settle for 10 min. To remove OMV-associated eDNA, 300–400 µg of OMV protein was treated with 4 units of TURBO DNaseI (Fisher Scientific) according to the manufacturer’s protocol. Briefly, after adding DNaseI and compatible reaction buffer, samples were incubated for 30 min at 37°C (200 rpm shaking) and pelleted by ultracentrifugation at 150,000×*g* for 2 h at 4°C. Pellets were resuspended in a 50 mM HEPES buffer (pH 6.8). The presence of eDNA on OMVs was verified by gel-electrophoresis and DNA staining. To analyze the impact of OMVs on biofilm formation, overnight-grown bacterial cultures were diluted in LB media to OD_600_ 0.02, and 250 µL of the corresponding culture was added to the well and slowly mixed with the OMVs by pipetting. Biofilms were allowed to grow for 6 h at 30°C statically. Images of the biofilms were obtained on a Zeiss LSM 880 confocal microscope at 20× magnification for image generation and subsequent biofilm parameters analysis. Imaris (Oxford Instruments) was used to process the images, and BiofilmQ (90) was used to measure the global biofilm parameters of the biofilms that formed in each test condition.

### Hydrophobicity analysis

Equal volumes of bacterial cultures grown overnight at 30°C (200 rpm shaking) were pelleted by centrifugation for 3 min at 15,000×*g*. Cell pellets were resuspended with 1× PBS (pH 7.4) to the final OD_600_ 0.5. Cell suspensions were mixed with the aliphatic petroleum hydrocarbon *n*-hexadecane (Sigma Aldrich) in a 3:1 ratio, vortexed for 30 s, and incubated statically at RT for 15 and 60 min. After the incubation, an OD_600_ of the aqueous phase was measured. The hydrophobicity was calculated as a percentage OD decrease upon incubation with *n*-hexadecane [(1 – (OD_600_ after incubation/OD_600_ before incubation)) * 100].

## SUPPLEMENTAL MATERIAL

Supplemental figures mbio.03304-23-s0001.pdf Figures 1 to 7.

Table S1 mbio.03304-23-s0002.pdf Matrix proteome.

Table S2 mbio.03304-23-s0003.pdf OMV proteome.

Table S3 mbio.03304-23-s0004.pdf ompU-WT proteome comparison.

Table S4 mbio.03304-23-s0005.pdf Strains and plasmids.

ASM does not own the copyrights to Supplemental Material that may be linked to, or accessed through, an article. The authors have granted ASM a non-exclusive, world-wide license to publish the Supplemental Material files. Please contact the corresponding author directly for reuse.
