# Supplementary material for: Outer membrane vesicles and the outer membrane protein OmpU govern Vibrio cholerae biofilm matrix assembly
Source: mBio. 2024 Jan 11;15(2):e03304-23. doi: 10.1128/mbio.03304-23 (PMC10865864; doi:10.1128/mbio.03304-23)
Supplement: Supplemental figures — Figures 1 to 7. [file mbio.03304-23-s0001.pdf]

Supplementary Figures

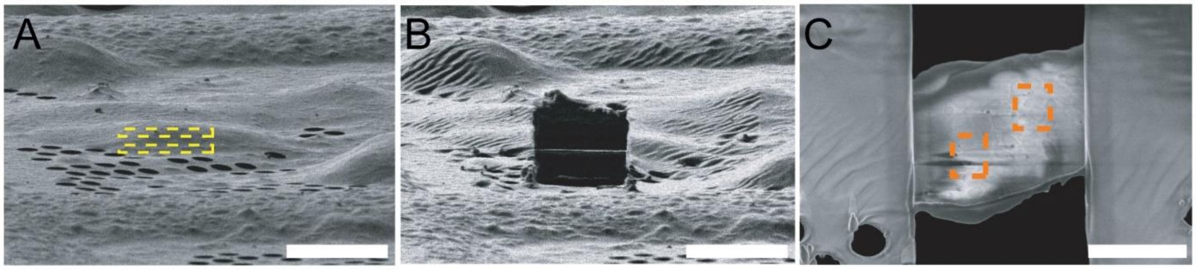

Figure S1. (A) Ion-beam image of a plunge-frozen biofilm sample prior to cryo-FIB milling. Yellow rectangles indicate the site of milling. **(B)** Ion-beam image of the same biofilm sample in A) after cryo-FIB milling. A thin lamella can be seen in the center of a cavity produced during the FIB milling. Scale bars in A and B = 10 μm. **(C)** Scanning electron microscopy (SEM) image showing the top view of the resulting lamella which displays a thin slice of cells within the biofilm. Orange rectangles with dashed lines indicate the sites of subsequent cryo-ET data collection. Scale bar = 5 μm.

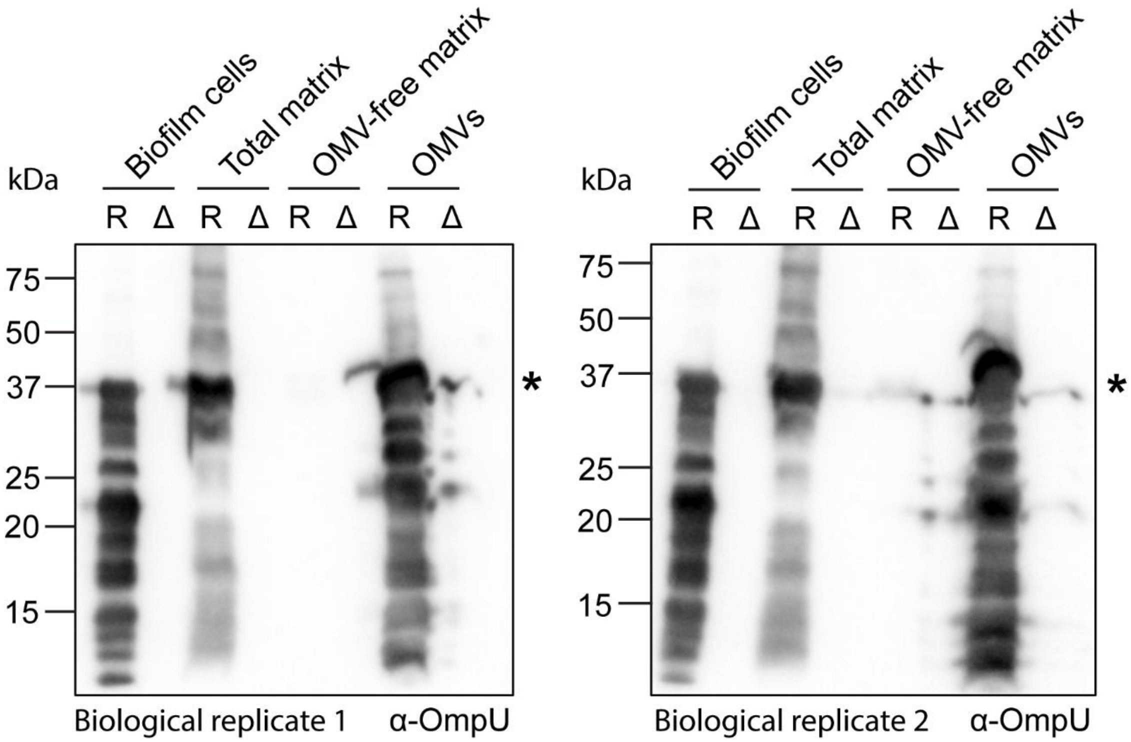

Figure S2. Representative immunoblots showing abundance of OmpU in biofilm cells and different components of biofilm matrix of Rugose (R) and Δ*ompU* (Δ) strains. Blots were probed against OmpU (predicted size is 36.7 kDa). Protein ladder sizes in kDa are indicated on the left side of each immunoblot.

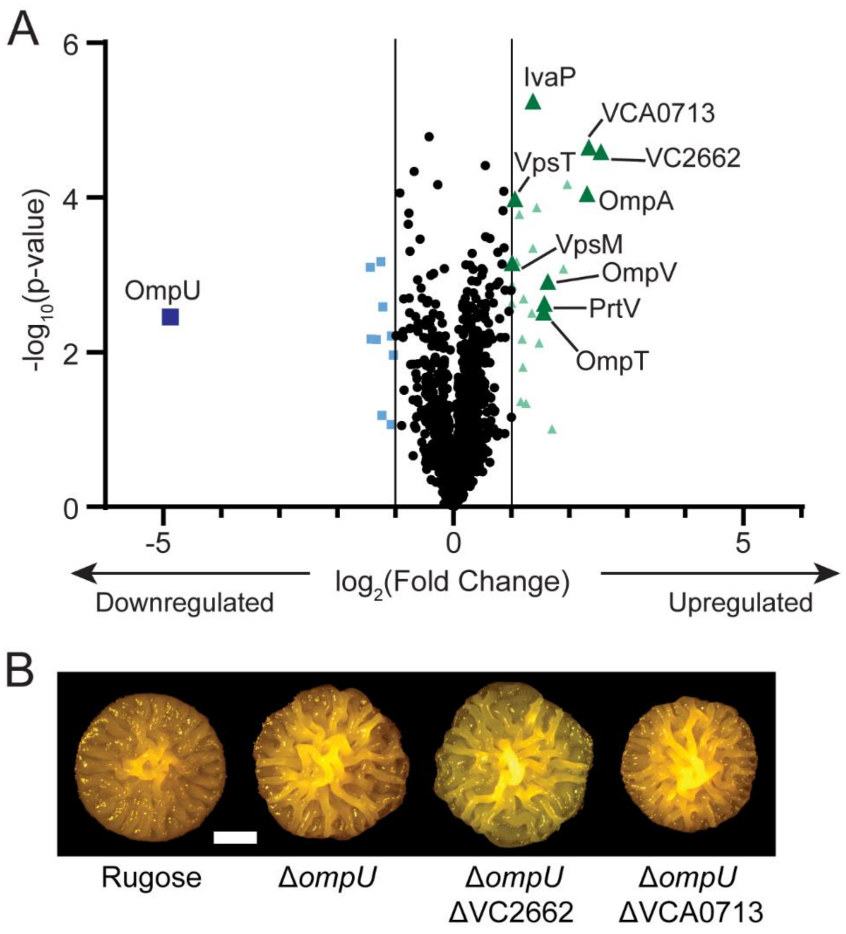

Figure S3. (A) Whole cell proteome comparison of rugose and Δ*ompU* strains. Volcano plot shows upregulated (green) and downregulated (blue) proteins in Δ*ompU* relative to the rugose parent strain. TMT-labeling followed by LC/MS-MS were used for the proteomic analysis of spot biofilms grown for 48h at 30°C. Experiment was performed with three biological replicates. **(B)** Colony morphology analysis of strains of indicated strains. Colonies were imaged after 72h growth at 30°C on LB agar plate. Scale bar = 1 mm.

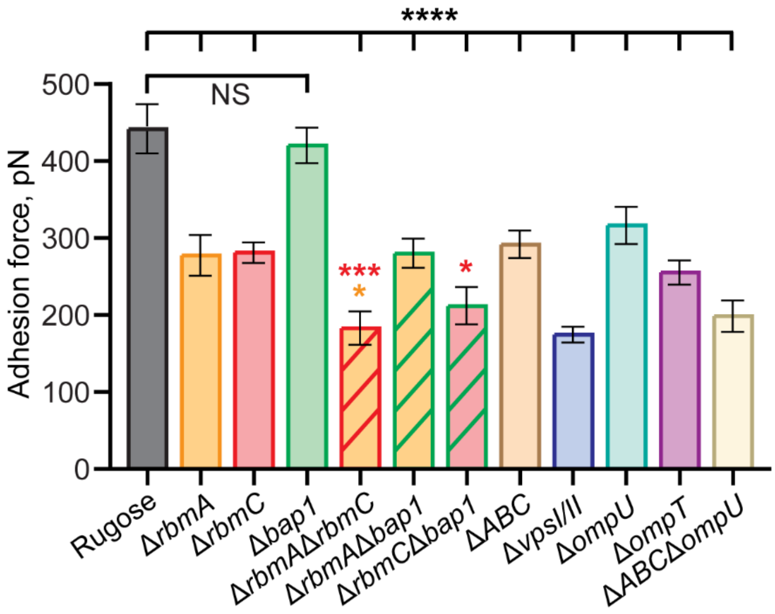

Figure S4. Average adhesion force for the indicated *V. cholerae* strains. Error bars represent standard error of the mean. Asterisks indicate the results of Student's T-test relative to the rugose parent strain and of the double deletion mutants relative to the corresponding single deletion strains (****: $p < 0.00001$, ***: $p < 0.0001$, ***: $p < 0.01$, NS = not significant).

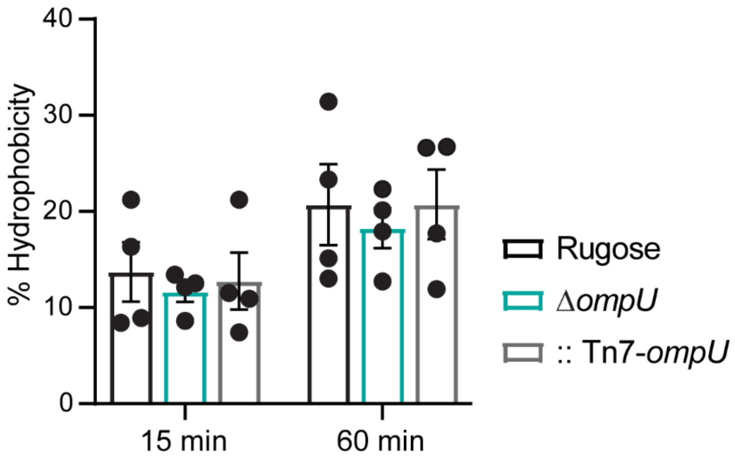

Figure S5. Analysis of cell surface hydrophobicity calculated as percentage of adhesion to hydrocarbons (N-hexadecane) for strains of indicated phenotypes. Error bars represent standard error of the mean.

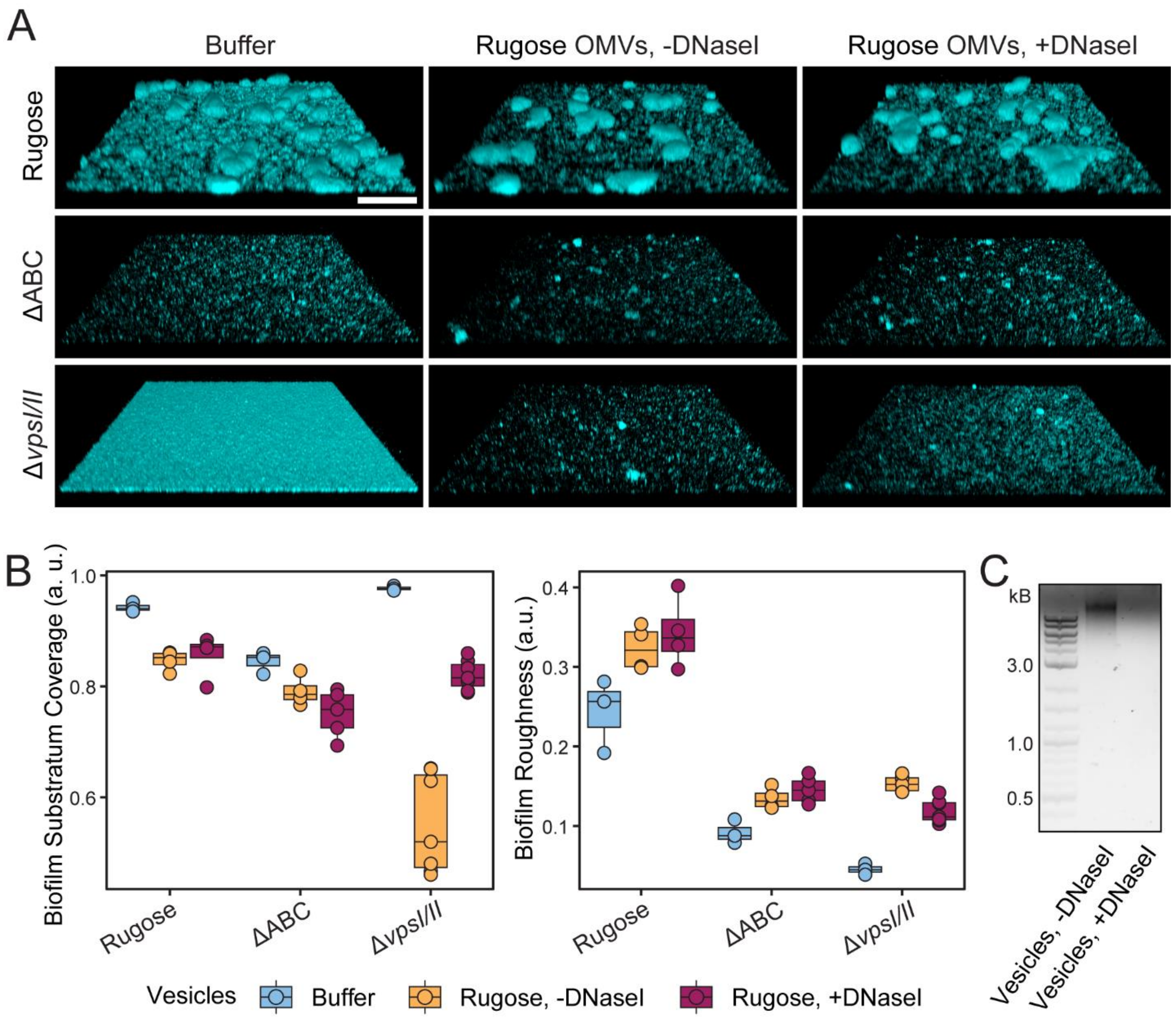

Figure S6. (A) Representative three-dimensional images of the static biofilms formed after 6h growth at 30°C by strains indicated on the left supplemented with the buffer (first column), rugose OMVs before and after DNaseI treatment. Scale bar = 100 μm. **(B)** Boxplot representation of the biofilm parameters of the images shown in Fig. S6.A analyzed using BiofilmQ software. **(C)** Agarose gel visualization eDNA associated with OMVs isolated from rugose strain before (left lane) and after (right lane) DNaseI treatment. DNA ladder sizes are indicated on the left side of the gel.

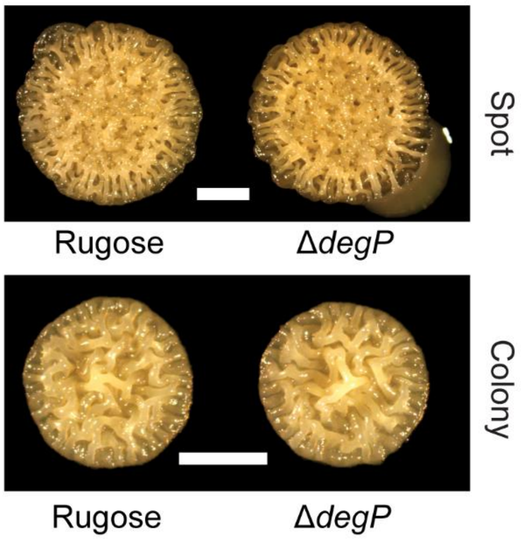

Figure S7. Spot and colony corrugation phenotypes of strains with indicated strains after 72 hours of growth on LB agar plates at 30°C. Scale bars = 2 mm.
