# Supplementary material for: Outer membrane vesicles and the outer membrane protein OmpU govern Vibrio cholerae biofilm matrix assembly
Source: mBio. 2024 Jan 11;15(2):e03304-23. doi: 10.1128/mbio.03304-23 (PMC10865864; doi:10.1128/mbio.03304-23)
Supplement: Table S1 — Matrix proteome. [file mbio.03304-23-s0002.pdf]

Supplementary Table 1. List of all proteins identified in biofilm matrix proteome

Label	Description	PsortB	Total spectral counts				
			M1	M2	M3	M4	M5
**VCA0865**	Hemagglutinin/proteinase	Extracellular	4511	4701	5022	6031	5774
**VC0633**	Outer membrane protein U	Outer Membrane	2310	1980	1900	1889	2057
**VCA0812**	Leucine aminopeptidase-related protein	Extracellular	1553	1479	1885	2178	1968
**VCA0813**	Aminopeptidase	Extracellular	1241	1326	1718	1964	1787
**VC1888**	Hemolysin-related protein	Extracellular	1055	998	1322	1282	1381
**VC1091**	Oligopeptide ABC transporter, periplasmic oligopeptide-binding protein	Periplasmic	915	841	918	1133	1079
**VCA0223**	Pre-pro-metalloprotease PrtV	Extracellular	807	796	1007	991	978
**VC1362**	Amino acid ABC transporter, periplasmic amino acid-binding protein	Periplasmic	648	718	926	936	1143
**VC0930**	Hemolysin-related protein	Extracellular	615	569	737	719	807
**VC2187**	Flagellin C	Extracellular	492	422	457	505	555
**VC2143**	Flagellin D	Extracellular	445	396	364	472	563
**VC0157**	Alkaline serine protease	Extracellular	425	421	446	496	412
**VC2142**	Flagellin B	Extracellular	372	361	398	432	550
**VC0171**	Peptide ABC transporter, periplasmic peptide-binding protein	Periplasmic	397	420	368	417	434
**VCA0883**	Non-hemolytic enterotoxin lytic component L1	Extracellular	250	221	251	325	400
**VC1952**	Chitinase	Extracellular	219	211	330	315	358
**VCA0738**	Conjugal transfer protein TraF	Unknown	332	285	234	192	246
**VC1929**	C4-dicarboxylate-binding periplasmic protein DctP	Periplasmic	196	156	300	276	329
**VC2738**	Phosphoenolpyruvate carboxykinase (ATP)	Cytoplasmic	226	203	229	253	302
**VC0608**	Iron(III) ABC transporter, periplasmic iron-compound-binding protein	Periplasmic	198	187	253	227	269
**VC0430**	Immunogenic protein	Unknown	196	157	201	223	264
**VC2188**	Flagellin A	Extracellular	163	166	175	210	240
**VCA0027**	Chitinase	Unknown	93	170	201	207	213
**VC0409**	MSHA pilin protein MshA	Unknown	154	133	126	123	130
**VCA0539**	UPF0312 protein VC_A0539	Unknown	69	61	160	145	221
**VC1425**	Putrescine-binding periplasmic protein	Periplasmic	120	112	128	115	155
**VC2000**	Glyceraldehyde-3-phosphate dehydrogenase	Cytoplasmic	81	105	146	139	150
**VC2412**	Dihydrolipoyl dehydrogenase	Cytoplasmic	121	113	129	107	118
**VC1141**	Isocitrate dehydrogenase [NADP]	Cytoplasmic	108	96	106	109	143
**VC2562**	2`,3`-cyclic-nucleotide 2`-phosphodiesterase	Periplasmic	100	108	113	97	117
**VC1485**	DUF3466 family protein	Unknown	107	115	96	102	95
**VCA0881**	Uncharacterized protein	Unknown	83	75	108	109	135
**VC1325**	Autoinducer 2-binding periplasmic protein LuxP	Periplasmic	106	80	88	73	141
**VC0034**	Thiol:disulfide interchange protein DsbA	Periplasmic	92	88	107	91	107
**VC2618**	Acetylornithine aminotransferase	Cytoplasmic	85	98	84	96	106
**VCA1028**	Maltoporin	Outer Membrane	151	106	66	58	45
**VC0336**	2,3-bisphosphoglycerate-independent phosphoglycerate mutase	Cytoplasmic	81	80	67	80	96
**VC0475**	Iron-regulated outer membrane virulence protein	Outer Membrane	75	79	79	79	89
**VC1863**	Amino acid ABC transporter, periplasmic amino acid-binding protein	Periplasmic	45	47	98	83	125
**VCA0981**	Solute-binding protein family 3/N-terminal domain-containing protein	Unknown	70	56	96	75	98
**VC1334**	Tricarboxylic transport TctC	Periplasmic	65	60	72	67	88

**VCA0685**	Iron(III) ABC transporter, periplasmic iron-compound-binding protein	Periplasmic	59	54	72	56	109
**VCA0576**	Heme transport protein HutA	Outer Membrane	90	67	56	47	76
**VC1560**	Catalase-peroxidase	Cytoplasmic	62	77	58	64	71
**VCA1113**	Putrescine-binding periplasmic protein	Periplasmic	58	54	62	68	70
**VC1523**	PBP domain-containing protein	Unknown	66	58	51	59	77
**VC1854**	Porin, putative	Outer Membrane	95	60	50	47	48
**VC2670**	Triosephosphate isomerase	Cytoplasmic	51	44	61	59	84
**VC2211**	Vibriobactin receptor	Outer Membrane	97	42	46	53	58
**VC0194**	Glutathione hydrolase proenzyme	Periplasmic	67	73	43	60	47
**VCA0945**	Maltodextrin-binding protein	Periplasmic	49	47	54	50	86
**VC2045**	Superoxide dismutase	Periplasmic	32	39	71	64	78
**VC0354**	Peptidyl-prolyl cis-trans isomerase	Periplasmic	55	58	57	56	56
**VC0432**	Malate dehydrogenase	Unknown	46	41	55	55	81
**VCA0645**	VOC domain-containing protein	Unknown	39	47	49	56	84
**VC1414**	Metal-dependent carboxypeptidase	Cytoplasmic	35	52	46	60	81
**VC2197**	Flagellar hook protein FlgE	Extracellular	70	57	39	54	47
**VC2350**	Deoxyribose-phosphate aldolase	Cytoplasmic	40	63	50	53	59
**VC0478**	Fructose-bisphosphate aldolase	Cytoplasmic	44	43	61	50	61
**VC2615**	DUF1338 domain-containing protein	Cytoplasmic	42	45	42	49	77
**VCA0759**	Arginine ABC transporter, periplasmic arginine-binding protein	Periplasmic	50	48	51	43	51
**VC1424**	Putrescine-binding periplasmic protein	Periplasmic	50	43	48	53	49
**VCA0900**	Metallo-beta-lactamase domain-containing protein	Unknown	44	47	41	51	56
**VC0928**	RbmA protein	Unknown	60	41	40	38	60
**VC2213**	Outer membrane protein OmpA	Outer Membrane	79	24	31	28	73
**VC1894**	Penicillin-binding protein activator LpoB	Unknown	67	33	45	29	59
**VCA1041**	Phosphomannomutase, putative	Unknown	42	40	36	40	68
**VCA1039**	Amino acid ABC transporter, periplasmic amino acid-binding protein	Periplasmic	40	46	45	44	51
**VCA0227**	Iron(III) ABC transporter, periplasmic iron-compound-binding protein	Periplasmic	52	44	44	38	46
**VCA0884**	Non-hemolytic enterotoxin lytic component L1	Inner Membrane	35	38	44	45	56
**VC2279**	Aminoacyl-histidine dipeptidase	Cytoplasmic	25	28	52	46	64
**VC2694**	Superoxide dismutase	Periplasmic	22	21	38	46	71
**VC1950**	trimethylamine-N-oxide reductase	Periplasmic	33	32	46	40	43
**VCA0882**	Uncharacterized protein	Inner Membrane	22	29	46	37	56
**VC1663**	Heat shock protein HslJ	Unknown	62	20	34	35	37
**VC2174**	5'-nucleotidase	Periplasmic	37	35	35	38	42
**VC1621**	Agglutination protein	Outer Membrane	44	38	27	36	40
**VC1154**	Uncharacterized protein	Unknown	77	35	18	24	30
**VC0968**	Cysteine synthase	Cytoplasmic	44	46	28	34	31
**VC1318**	Outer membrane protein OmpV	Outer Membrane	69	59	18	11	23
**VCA0863**	Lysophospholipase VolA	Unknown	39	45	24	32	38
**VC2095**	Phosphoglucomutase	Cytoplasmic	34	41	30	33	31
**VCA0623**	Transaldolase	Cytoplasmic	28	30	27	30	49
**VCA0877**	Hydrolase, putative	Unknown	27	41	27	31	38
**VC2144**	Flagellin E	Extracellular	34	31	26	34	30
**VC2550**	YtfJ family protein	Inner Membrane	30	29	27	31	37

**VCA0140**	Spindolin-related protein	Unknown	33	27	30	32	27
**VC0298**	Acetyl-coenzyme A synthetase	Cytoplasmic	23	32	27	23	41
**VC0244**	Perosamine synthase	Cytoplasmic	31	28	23	30	33
**VC0374**	Glucose-6-phosphate isomerase	Cytoplasmic	22	30	23	26	32
**VCA0925**	Dihydroorotase	Cytoplasmic	23	28	26	25	30
**VC0028**	Dihydroxy-acid dehydratase	Cytoplasmic	22	31	26	27	23
**VC0604**	Aconitate hydratase B	Cytoplasmic	27	23	22	21	36
**VC0964**	PTS system, glucose-specific IIA component	Cytoplasmic	33	22	18	20	33
**VC2036**	Aspartate-semialdehyde dehydrogenase 1	Cytoplasmic	24	19	23	29	27
**VC0620**	Peptide ABC transporter, periplasmic peptide-binding protein	Periplasmic	23	22	21	23	31
**VCA0849**	RTX toxin	Extracellular	24	26	21	25	23
**VC1384**	Outer membrane protein beta-barrel domain-containing protein	Unknown	23	14	24	25	30
**VCA1033**	Extracellular solute-binding protein, putative	Periplasmic	29	30	19	19	17
**VCA0513**	Amino acid biosynthesis aminotransferase	Cytoplasmic	28	24	21	17	24
**VC1346**	Fumarylacetoacetate hydrolase family protein	Unknown	24	19	17	24	30
**VC1836**	Tol-Pal system protein TolB	Periplasmic	28	17	23	19	24
**VC2230**	Phosphoheptose isomerase	Cytoplasmic	21	21	22	19	25
**VCA0867**	Outer membrane protein W	Outer Membrane	36	42	11	12	5
**VC1034**	Uridine phosphorylase	Cytoplasmic	19	20	19	24	22
**VCA0978**	Amino acid ABC transporter, periplasmic amino acid-binding protein, putative	Periplasmic	15	17	22	22	22
**VCA0197**	GMP reductase	Cytoplasmic	26	22	16	18	14
**VC0581**	Penicillin-binding protein activator LpoA	Inner Membrane	33	11	5	12	30
**VCA0568**	Conjugal transfer protein TraF	Outer Membrane	27	16	10	10	25
**VC1293**	Aminotransferase	Cytoplasmic	20	20	13	12	21
**VC0736**	Isocitrate lyase	Cytoplasmic	20	17	15	15	19
**VCA0702**	Iron-containing alcohol dehydrogenase	Cytoplasmic	11	13	19	15	27
**VC0156**	Vitamin B12 transporter BtuB	Outer Membrane	22	13	17	14	17
**VC1983**	Peptidase, putative	Cytoplasmic	17	14	14	19	19
**VC2542**	UDP-N-acetylmuramate--L-alanyl-gamma-D-glutamyl-meso-2,6-diaminoheptandioate ligase	Cytoplasmic	13	10	21	15	20
**VCA0975**	endopeptidase La	Cytoplasmic	18	18	9	14	19
**VC1010**	Probable lactoylglutathione lyase	Cytoplasmic	12	4	19	24	17
**VC2002**	DUF2860 domain-containing protein	Outer Membrane	15	12	17	15	16
**VC2290**	Na(+)-translocating NADH-quinone reductase subunit F	Cytoplasmic	11	16	19	16	11
**VCA0037**	Copper chaperone PCu(A)C	Unknown	18	9	9	18	19
**VCA0886**	2-amino-3-ketobutyrate coenzyme A ligase	Cytoplasmic	15	10	12	15	19
**VC1895**	DUF1425 domain-containing protein	Unknown	19	16	7	10	19
**VC2347**	Purine nucleoside phosphorylase DeoD-type 1	Cytoplasmic	14	7	15	14	19
**VC0755**	Peptidase B	Cytoplasmic	9	20	13	15	12
**VC2168**	DUF2066 domain-containing protein	Unknown	10	9	15	15	20
**VC2190**	Flagellar hook-associated protein FlgL	Extracellular	24	13	10	8	13
**VC2157**	4-hydroxy-tetrahydrodipicolinate synthase	Cytoplasmic	11	13	15	11	17
**VC2305**	Outer membrane protein OmpK	Outer Membrane	9	10	15	13	18
**VC1048**	Putative NAD(P)H nitroreductase	Unknown	13	10	10	6	26
**VC0734**	Malate synthase	Cytoplasmic	8	14	11	16	16
**VCA0130**	Autoinducer 2-binding periplasmic protein LuxP	Periplasmic	13	11	10	16	14
**VC2299**	Peptidyl-prolyl cis-trans isomerase	Periplasmic	12	9	15	14	14

**VC0446**	LPS-assembly protein LptD	Outer Membrane	15	14	11	10	13
**VC2447**	Enolase	Cytoplasmic	11	8	14	12	15
**VC2099**	Flavodoxin	Cytoplasmic	14	11	6	11	18
**VC2501**	Cytosol aminopeptidase	Cytoplasmic	12	11	11	12	13
**VCA0328**	Biphenyl-2,3-diol 1,2-dioxygenase III-related protein	Cytoplasmic	8	7	15	12	16
**VCA0591**	Peptide ABC transporter, periplasmic peptide-binding protein, putative	Periplasmic	15	10	11	10	12
**VC2436**	Outer membrane protein TolC	Outer Membrane	20	8	7	9	13
**VC1494**	Aminopeptidase N	Cytoplasmic	12	12	11	9	12
**VC0483**	Oxidative stress defense protein	Periplasmic	11	14	7	13	11
**VCA0144**	Immunogenic protein	Unknown	5	6	11	12	21
**VCA0811**	GlcNAc-binding protein A	Extracellular	10	13	9	13	9
**VCA0690**	Acetyl-CoA acetyltransferase	Cytoplasmic	12	11	6	12	13
**VC0362**	Elongation factor Tu-B	Cytoplasmic	14	5	14	12	8
**VC2191**	Flagellar hook-associated protein 1	Extracellular	18	8	6	6	15
**VC0372**	Alanine racemase	Cytoplasmic	13	7	10	8	14
**VC0776**	Ferric vibriobactin ABC transporter, periplasmic ferric vibriobactin-binding protein	Periplasmic	11	9	12	9	10
**VC2252**	Outer membrane protein assembly factor BamA	Outer Membrane	13	9	9	8	12
**VC1866**	Formate acetyltransferase	Cytoplasmic	8	13	8	11	11
**VC1064**	Lipoprotein-related protein	Unknown	13	9	6	12	10
**VC0538**	Thiosulfate ABC transporter, periplasmic thiosulfate-binding protein	Periplasmic	6	6	9	6	21
**VC0186**	Glutathione reductase	Cytoplasmic	7	10	9	9	13
**VCA0678**	Periplasmic nitrate reductase	Periplasmic	18	12	8	5	4
**VCA0207**	NH(3)-dependent NAD(+) synthetase	Cytoplasmic	6	8	9	11	13
**VC1101**	Uncharacterized protein	Unknown	11	6	8	5	17
**VC1933**	Oxidored_molyb domain-containing protein	Inner Membrane	5	7	7	13	14
**VC0667**	Oxidoreductase Tas, aldo/keto reductase family	Cytoplasmic	9	6	7	10	13
**VCA0624**	Transketolase 2	Cytoplasmic	8	7	10	9	10
**VC0414**	DUF6701 domain-containing protein	Unknown	8	9	11	11	5
**VC2616**	N-succinylglutamate 5-semialdehyde dehydrogenase	Cytoplasmic	6	11	6	8	12
**VC0972**	Porin, putative	Outer Membrane	7	6	8	6	16
**VC2261**	Methionine aminopeptidase	Cytoplasmic	7	3	8	10	14
**VC1779**	Sialic acid-binding periplasmic protein SiaP	Periplasmic	7	5	9	7	13
**VC1098**	Acetate kinase 1	Cytoplasmic	6	9	13	5	8
**VC0756**	Nucleoside diphosphate kinase	Cytoplasmic	10	6	9	7	9
**VCA0828**	Phenylalanine-4-hydroxylase	Cytoplasmic	6	6	9	10	9
**VCA0136**	Glycerophosphoryl diester phosphodiesterase	Periplasmic	5	4	7	10	14
**VC1554**	Glycerophosphoryl diester phosphodiesterase, putative	Cytoplasmic	9	10	7	6	8
**VC0772**	Vibriobactin-specific 2,3-dihydroxybenzoate-AMP ligase	Cytoplasmic	4	5	9	6	14
**VCA0625**	TonB receptor-related protein	Outer Membrane	10	7	9	7	5
**VC0905**	Probable D-methionine-binding lipoprotein MetQ	Inner Membrane	7	6	6	9	9
**VC0762**	Outer membrane protein assembly factor BamB	Outer Membrane	5	4	12	8	8
**VC1255**	ribonucleoside-diphosphate reductase	Cytoplasmic	3	5	8	8	13
**VC1195**	Lipoprotein, putative	Outer Membrane	8	9	5	7	8
**VCA0829**	Acetyl-CoA synthase	Cytoplasmic	7	7	4	10	8
**VC0306**	Thioredoxin	Cytoplasmic	8	6	7	7	8
**VC0188**	oligopeptidase A	Cytoplasmic	4	9	6	8	8

**VC0477**	Phosphoglycerate kinase	Cytoplasmic	6	0	3	6	19
**VC0120**	Porphobilinogen deaminase	Cytoplasmic	5	3	8	9	9
**VC1184**	NifS-related protein	Cytoplasmic	10	6	4	4	10
**VC0537**	Cysteine synthase	Cytoplasmic	10	6	4	6	7
**VC2210**	Vibriobactin utilization protein ViuB	Cytoplasmic	4	7	6	4	11
**VC2491**	3-isopropylmalate dehydrogenase	Cytoplasmic	10	5	4	6	7
**VC1344**	4-hydroxyphenylpyruvate dioxygenase	Cytoplasmic	6	3	8	10	5
**VC0058**	Carbonic anhydrase, family 3	Cytoplasmic	6	7	7	6	6
**VC1704**	5-methyltetrahydropteroyltriglutamate--homocysteine methyltransferase	Cytoplasmic	7	7	3	5	9
**VC0132**	Uncharacterized protein	Unknown	9	3	4	6	9
**VC2298**	Lipoprotein, putative	Unknown	6	2	8	5	9
**VC0275**	Phosphoribosylamine--glycine ligase	Cytoplasmic	7	4	6	5	7
**VCA0897**	6-phosphogluconolactonase	Unknown	6	4	6	5	8
**VCA0862**	Long-chain fatty acid transport protein	Outer Membrane	9	8	2	4	6
**VCA0838**	OsmC/Ohr family protein	Unknown	4	4	4	8	9
**VCA0018**	Type VI secretion system spike protein VgrG2	Cytoplasmic	4	7	8	5	4
**VC1942**	Bifunctional protein FolD	Cytoplasmic	4	5	6	6	7
**VCA0219**	Hemolysin	Extracellular	6	6	5	7	3
**VC2268**	6,7-dimethyl-8-ribityllumazine synthase	Cytoplasmic	6	2	5	9	5
**VC0013**	Beta sliding clamp	Cytoplasmic	4	7	5	5	6
**VCA0700**	Chitodextrinase	Periplasmic	5	7	5	6	4
**VC2535**	PmbA protein	Unknown	4	5	6	8	4
**VC2480**	Ribose-5-phosphate isomerase A	Cytoplasmic	9	6	1	5	5
**VC1182**	Thioredoxin reductase	Unknown	6	6	8	3	3
**VC2539**	Thiamine-binding periplasmic protein	Periplasmic	7	10	2	4	3
**VC2270**	Riboflavin synthase, alpha subunit	Cytoplasmic	7	4	4	5	6
**VC1416**	Actin cross-linking toxin VgrG1	Cytoplasmic	3	6	4	7	5
**VCA0691**	Acetoacetyl-CoA reductase	Cytoplasmic	7	8	5	3	2
**VC0935**	Capsular polysaccharide synthesis enzyme CpsB	Outer Membrane	6	3	5	6	5
**VC0573**	Ubiquinol-cytochrome c reductase iron-sulfur subunit	Inner Membrane	4	5	6	4	6
**VC1532**	N-acetyl-D-glucosamine kinase	Cytoplasmic	1	4	9	4	6
**VC0332**	Uroporphyrinogen decarboxylase	Cytoplasmic	6	7	4	4	3
**VC2669**	5-carboxymethyl-2-hydroxymuconate delta isomerase, putative	Cytoplasmic	5	4	6	3	6
**VC2111**	NADPH-dependent FMN reductase-like domain-containing protein	Unknown	5	6	6	4	3
**VC0675**	Thymidylate synthase	Cytoplasmic	6	3	7	3	4
**VC1134**	Histidinol-phosphate aminotransferase	Cytoplasmic	4	5	5	2	7
**VC2145**	TyrA protein	Cytoplasmic	4	2	3	3	11
**VC1664**	ABC transporter, periplasmic substrate-binding protein, putative	Periplasmic	8	6	1	4	4
**VC1200**	Trypsin, putative	Unknown	5	5	4	5	4
**VC0146**	Ribosomal RNA small subunit methyltransferase D	Cytoplasmic	9	7	1	2	4
**VCA0885**	L-threonine 3-dehydrogenase	Cytoplasmic	6	3	5	3	5
**VC2379**	5'-methylthioadenosine/S-adenosylhomocysteine nucleosidase	Cytoplasmic	4	2	3	4	9
**VC1203**	Urocanate hydratase	Cytoplasmic	5	5	2	4	6
**VCA1077**	NirV	Unknown	5	4	2	5	6
**VCA0935**	Uncharacterized protein	Unknown	6	7	1	3	5

**VC1492**	glutamate dehydrogenase	Cytoplasmic	5	3	8	5	1
**VC1483**	3-hydroxydecanoyl-[acyl-carrier-protein] dehydratase	Cytoplasmic	5	7	1	3	5
**VCA0610**	Glyoxalase	Cytoplasmic	3	4	5	5	4
**VC0576**	Stringent starvation protein A	Cytoplasmic	5	6	3	3	4
**VC1730**	DNA topoisomerase 1	Cytoplasmic	6	2	2	2	8
**VC0556**	Glutamate--cysteine ligase	Cytoplasmic	3	2	4	3	8
**VC1819**	Aldehyde dehydrogenase	Cytoplasmic	7	3	3	5	2
**VC0067**	Aminopeptidase P	Cytoplasmic	3	3	4	4	6
**VCA0337**	Putative carboxypeptidase VC_A0337	Cytoplasmic	5	2	2	3	7
**VC1702**	YCII-related domain-containing protein	Unknown	5	3	2	3	6
**VC1622**	Outer membrane protein, putative	Outer Membrane	3	3	4	5	4
**VC1299**	6-carboxy-5,6,7,8-tetrahydropterin synthase	Cytoplasmic	4	3	5	4	3
**VCA0711**	Methylglyoxal synthase	Cytoplasmic	3	2	6	5	2
**VC1834**	Cell division coordinator CpoB	Unknown	4	3	4	2	5
**VC1183**	Agglutination protein	Cytoplasmic	5	1	4	2	6
**VC2645**	Acetylornithine deacetylase	Cytoplasmic	3	1	3	4	6
**VC0361**	Elongation factor G 1	Cytoplasmic	4	4	2	2	5
**VCA0734**	OMP_b-brl domain-containing protein	Unknown	4	0	3	4	6
**VC1835**	Peptidoglycan-associated lipoprotein	Outer Membrane	4	2	4	3	4
**VC1672**	DNA-3-methyladenine glycosidase I	Unknown	2	6	3	3	3
**VC1603**	Solute-binding protein family 3/N-terminal domain-containing protein	Unknown	4	3	3	3	4
**VC1583**	Superoxide dismutase [Cu-Zn]	Periplasmic	4	2	3	3	5
**VC0973**	VvgS protein	Unknown	6	3	4	2	2
**VC0566**	Periplasmic serine endoprotease DegP-like	Periplasmic	5	3	1	4	4
**VC0488**	Extracytoplasmic solute receptor protein	Unknown	5	4	5	2	1
**VC0139**	DPS family protein	Cytoplasmic	4	2	2	5	4
**VC0029**	Branched-chain-amino-acid aminotransferase	Cytoplasmic	2	3	5	3	4
**VC2764**	ATP synthase subunit beta	Cytoplasmic	8	2	2	3	1
**VC1732**	3-phosphoshikimate 1-carboxyvinyltransferase	Cytoplasmic	3	1	2	4	6
**VC0341**	Oligoribonuclease	Cytoplasmic	9	2	1	1	3
**VC2432**	Esterase YqiA	Cytoplasmic	2	1	3	5	5
**VC2514**	UDP-N-acetylglucosamine 1-carboxyvinyltransferase	Cytoplasmic	6	2	2	3	2
**VCA1056**	Methyl-accepting chemotaxis protein	Inner Membrane	5	2	2	1	5
**VC1350**	Glutathione-dependent peroxiredoxin	Unknown	7	3	1	1	3
**VC0578**	Hemolysin, putative	Periplasmic	4	5	1	2	3
**VC0174**	SPOR domain-containing protein	Periplasmic	3	4	1	4	3
**VC1133**	Histidinol dehydrogenase	Cytoplasmic	2	8	1	3	0
**VC2251**	Chaperone protein Skp	Periplasmic	5	1	1	2	5
**VC1312**	Broad specificity amino-acid racemase	Unknown	3	4	4	0	3
**VC2198**	Basal-body rod modification protein FlgD	Extracellular	3	5	1	1	4
**VC1872**	PrkA AAA domain-containing protein	Cytoplasmic	3	1	4	5	1
**VC0731**	Alkyl hydroperoxide reductase C	Cytoplasmic	4	1	3	5	1
**VC1202**	Histidine ammonia-lyase	Cytoplasmic	2	1	3	3	4
**VC0708**	Outer membrane protein assembly factor BamD	Outer Membrane	3	2	2	3	3
**VC0055**	Oxygen-dependent coproporphyrinogen-III oxidase	Cytoplasmic	3	1	2	3	4
**VCA0859**	Oxidoreductase, aldo/keto reductase 2 family	Cytoplasmic	1	4	2	3	3
**VCA0593**	Exopolyphosphatase-related protein	Cytoplasmic	2	1	1	2	7

**VC2416**	2`,3`-cyclic-nucleotide 2`-phosphodiesterase, putative	Periplasmic	3	3	1	2	4
**VC2059**	Purine-binding chemotaxis protein CheW	Cytoplasmic	1	2	5	4	1
**VC1849**	Peptidyl-prolyl cis-trans isomerase	Cytoplasmic	2	2	3	1	5
**VC1709**	Zinc protease, insulinase family	Unknown	2	3	3	3	2
**VCA0894**	Probable phosphatase VC_A0894	Cytoplasmic	2	3	2	3	2
**VC2366**	Putative 4-hydroxy-4-methyl-2-oxoglutarate aldolase	Cytoplasmic	1	3	3	2	3
**VC2621**	Extracellular nuclease-related protein	Unknown	2	1	4	2	3
**VC2517**	Phospholipid-binding protein MlaC	Unknown	1	3	0	2	6
**VC2433**	3',5'-cyclic adenosine monophosphate phosphodiesterase CpdA	Cytoplasmic	2	2	4	2	2
**VC2766**	ATP synthase subunit alpha	Cytoplasmic	2	1	5	3	0
**VCA0432**	VOC domain-containing protein	Unknown	0	0	2	3	6
**VC1887**	Peptidoglycan-binding protein CsiV	Unknown	3	0	2	3	3
**VC1042**	Long-chain fatty acid transport protein	Outer Membrane	3	1	3	2	2
**VC0767**	Inosine-5'-monophosphate dehydrogenase	Cytoplasmic	1	1	2	4	3
**VC0758**	HTH cro/C1-type domain-containing protein	Unknown	2	2	3	1	3
**VC0422**	TldD protein	Cytoplasmic	2	3	4	1	1
**VC0381**	Uncharacterized protein	Unknown	5	1	1	2	2
**VC0168**	Cytochrome c5	Periplasmic	0	1	3	2	5
**VC0476**	D-erythrose-4-phosphate dehydrogenase	Cytoplasmic	1	2	3	2	2
**VC2141**	Protein FlaG	Unknown	2	4	1	2	1
**VCA0053**	Purine nucleoside phosphorylase DeoD-type 2	Cytoplasmic	0	3	2	3	2
**VCA0026**	UPF0319 protein VC_A0026	Unknown	3	0	0	1	6
**VC1623**	Carboxynorspermidine/carboxyspermidine decarboxylase	Cytoplasmic	2	2	0	4	2
**VC0435**	50S ribosomal protein L21	Cytoplasmic	0	0	5	4	1
**VC0327**	50S ribosomal protein L7/L12	Unknown	5	1	1	1	2
**VCA0765**	L-allo-threonine aldolase	Cytoplasmic	1	2	3	2	2
**VCA0644**	NADH oxidase, putative	Unknown	2	3	2	1	2
**VC2524**	3-deoxy-D-manno-octulosonate 8-phosphate phosphatase KdsC	Cytoplasmic	1	5	0	1	3
**VC2422**	nicotinate-nucleotide diphosphorylase (carboxylating)	Cytoplasmic	2	1	2	2	3
**VC1059**	Oxidoreductase, short-chain dehydrogenase/reductase family	Cytoplasmic	2	0	0	4	4
**VC1049**	Transcriptional regulator, LysR family	Cytoplasmic	1	2	2	3	2
**VC0715**	NADPH-flavin oxidoreductase	Inner Membrane	2	0	2	2	4
**VC0710**	Purine nucleoside phosphorylase	Unknown	1	2	3	2	2
**VC0502**	Type IV pilin, putative	Unknown	2	0	3	3	2
**VC0408**	MSHA pilin protein MshB	Unknown	1	0	2	3	4
**VC2664**	Chaperonin GroEL 1	Cytoplasmic	2	1	3	2	1
**VC2359**	Uracil-DNA glycosylase	Cytoplasmic	2	1	2	1	3
**VC2019**	3-oxoacyl-[acyl-carrier-protein] synthase 2	Cytoplasmic	4	0	1	1	3
**VC0752**	Chaperone protein HscA homolog	Cytoplasmic	2	2	2	2	1
**VC0461**	Pyridoxal phosphate homeostasis protein	Cytoplasmic	1	1	2	2	3
**VC0445**	Chaperone SurA	Periplasmic	2	2	1	1	3
**VCA0913**	Hemin ABC transporter, periplasmic hemin-binding protein HutB	Periplasmic	1	1	2	1	4
**VCA0814**	Agmatinase	Cytoplasmic	0	0	5	1	3
**VCA0712**	Pyrazinamidase/nicotinamidase	Cytoplasmic	2	1	1	2	3
**VCA0581**	Peptidase M48 domain-containing protein	Unknown	2	1	1	2	3
**VC2568**	Peptidyl-prolyl cis-trans isomerase	Cytoplasmic	2	2	1	0	4

**VC2413**	Acetyltransferase component of pyruvate dehydrogenase complex	Cytoplasmic	4	2	1	1	1
**VC1703**	Uncharacterized protein	Unknown	3	0	1	1	4
**VC1369**	SsuA/THI5-like domain-containing protein	Inner Membrane	3	1	0	0	5
**VC0485**	Pyruvate kinase	Cytoplasmic	3	0	2	1	3
**VC0344**	N-acetylmuramoyl-L-alanine amidase	Unknown	2	2	2	1	2
**VC2628**	3-dehydroquinate synthase	Cytoplasmic	1	1	2	2	2
**VC2472**	tRNA-modifying protein YgfZ	Unknown	5	1	0	0	2
**VC2272**	Transcriptional repressor NrdR	Cytoplasmic	4	1	2	0	1
**VC0179**	Cyclic GMP-AMP synthase	Cytoplasmic	2	0	1	1	4
**VCA1016**	Lipoprotein	Unknown	1	2	2	1	2
**VCA0558**	Gamma-glutamyltranspeptidase, putative	Periplasmic	4	0	0	1	3
**VCA0459**	Lipoprotein	Unknown	1	1	3	1	2
**VCA0346**	H-REV 107-related protein	Unknown	2	1	2	1	2
**VCA0059**	Major outer membrane lipoprotein Lpp	Outer Membrane	4	0	1	1	2
**VC2418**	Thiol:disulfide interchange protein	Periplasmic	2	1	1	1	3
**VC2204**	Negative regulator of flagellin synthesis	Unknown	3	3	0	1	1
**VC2092**	Citrate synthase	Cytoplasmic	1	0	1	5	1
**VC2089**	Succinate dehydrogenase flavoprotein subunit	Inner Membrane	2	1	1	2	2
**VC1089**	Periplasmic binding protein-related protein	Periplasmic	1	0	2	2	3
**VC0554**	Protease, insulinase family/protease, insulinase family	Unknown	2	2	0	1	3
**VC0855**	Chaperone protein DnaK	Cytoplasmic	0	1	1	1	4
**VC0837**	Toxin coregulated pilus biosynthesis protein F	Unknown	2	1	2	1	1
**VC0851**	Outer membrane protein assembly factor BamE	Outer Membrane	1	1	2	2	1
**VCA0277**	Glycine cleavage system H protein	Cytoplasmic	2	0	2	1	2
**VC2736**	33 kDa chaperonin	Cytoplasmic	1	0	2	2	2
**VC2579**	30S ribosomal protein S5	Cytoplasmic	1	2	2	2	0
**VC1297**	Asparagine--tRNA ligase	Cytoplasmic	3	0	1	0	3
**VC0528**	2-C-methyl-D-erythritol 4-phosphate cytidylyltransferase	Cytoplasmic	1	0	2	3	1
**VC0384**	Sulfite reductase [NADPH] flavoprotein alpha-component	Inner Membrane	1	2	1	2	1
**VCA1075**	PEGA domain-containing protein	Unknown	0	0	1	1	5
**VCA0880**	Uncharacterized protein	Unknown	2	2	1	1	1
**VCA0632**	MutT/nudix family protein	Cytoplasmic	3	0	2	1	1
**VCA0345**	DUF1349 domain-containing protein	Unknown	1	2	2	1	1
**VC2625**	Ribulose-phosphate 3-epimerase	Cytoplasmic	1	3	1	0	2
**VC2456**	Uncharacterized protein	Outer Membrane	1	0	2	2	2
**VC2358**	Hemerythrin-like domain-containing protein	Cytoplasmic	3	0	0	1	3
**VC2004**	Haem-binding uptake Tiki superfamily ChaN domain-containing protein	Unknown	2	1	1	1	2
**VC1987**	Outer membrane lipoprotein Slp, putative	Outer Membrane	4	2	0	1	0
**VC1745**	Succinate-semialdehyde dehydrogenase	Cytoplasmic	0	1	1	3	2
**VC0373**	YjbQ family protein	Unknown	2	1	1	2	1
**VC0103**	Uncharacterized protein	Cytoplasmic	3	2	0	1	1
**VC0078**	Ferritin	Cytoplasmic	2	2	1	2	0
**VC0026**	Zinc-binding alcohol dehydrogenase	Cytoplasmic	2	1	2	1	1
**VC0017**	Uncharacterized protein	Unknown	4	0	1	2	0
**VC0545**	Alanine--tRNA ligase	Cytoplasmic	0	2	0	1	3
**VCA0580**	Putative esterase VC_A0580	Unknown	1	2	1	1	1
**VC2587**	30S ribosomal protein S17	Cytoplasmic	1	1	2	2	0

**VC2342**	Elongation factor G 2	Cytoplasmic	0	1	2	3	0
**VC2260**	30S ribosomal protein S2	Cytoplasmic	1	0	0	3	2
**VC2156**	Outer membrane protein assembly factor BamC	Outer Membrane	5	0	0	0	1
**VC1738**	Enoyl-[acyl-carrier-protein] reductase [NADH] 1	Cytoplasmic	1	0	1	1	3
**VC0570**	50S ribosomal protein L13	Cytoplasmic	1	1	2	2	0
**VCA0415**	VOC domain-containing protein	Unknown	1	1	0	1	3
**VC2527**	Lipopolysaccharide export system protein LptA	Unknown	3	0	1	1	1
**VC2362**	Threonine synthase	Cytoplasmic	1	1	1	2	1
**VC2196**	Flagellar basal-body rod protein FlgF	Periplasmic	0	0	0	2	4
**VC2081**	High-affinity zinc uptake system protein ZnuA	Periplasmic	1	1	2	0	2
**VC1938**	Thioesterase	Cytoplasmic	1	2	1	1	1
**VC0942**	Lipoprotein	Unknown	3	3	0	0	0
**VC0911**	Trehalose-6-phosphate hydrolase	Cytoplasmic	0	1	1	0	4
**VC2106**	Ferric uptake regulation protein	Cytoplasmic	1	2	0	1	1
**VCA0907**	Heme oxygenase HutZ	Cytoplasmic	3	0	0	1	1
**VC2458**	Pyridoxine 5'-phosphate synthase	Cytoplasmic	1	0	1	1	2
**VC2236**	Hydroxyacylglutathione hydrolase	Cytoplasmic	0	1	0	2	2
**VC2025**	50S ribosomal protein L32	Cytoplasmic	3	0	1	1	0
**VC1727**	Glucose-1-phosphate adenylyltransferase 1	Cytoplasmic	1	0	1	2	1
**VC1692**	Trimethylamine-N-oxide reductase	Periplasmic	0	0	2	2	1
**VC1508**	UPF0234 protein VC_1508	Cytoplasmic	2	0	1	1	1
**VC1345**	Putative dioxygenase VC_1345	Cytoplasmic	1	0	1	3	0
**VC0692**	Beta-hexosaminidase	Cytoplasmic	2	0	1	0	2
**VC0052**	N5-carboxyaminoimidazole ribonucleotide mutase	Unknown	1	0	1	1	2
**VCA1035**	Uncharacterized protein	Unknown	1	0	2	1	1
**VCA0958**	HTH cro/C1-type domain-containing protein	Cytoplasmic	1	0	1	1	2
**VCA0726**	Molybdenum ABC transporter, periplasmic molybdenum-binding protein	Periplasmic	2	2	0	0	1
**VCA0139**	DUF3316 domain-containing protein	Unknown	1	0	0	2	2
**VC2747**	DUF4124 domain-containing protein	Unknown	1	1	2	0	1
**VC2022**	Malonyl CoA-acyl carrier protein transacylase	Cytoplasmic	1	0	1	1	2
**VC1893**	Aminoglycoside phosphotransferase domain-containing protein	Cytoplasmic	1	0	2	1	1
**VC2720**	Fe/S biogenesis protein NfuA	Cytoplasmic	0	2	1	1	0
**VC2586**	50S ribosomal protein L14	Cytoplasmic	0	1	1	2	0
**VC2273**	Gamma-glutamyl phosphate reductase	Unknown	1	2	0	1	0
**VC2140**	Flagellar hook-associated protein 2	Extracellular	2	1	1	0	0
**VC0986**	Adenylate kinase	Cytoplasmic	1	0	1	1	1
**VC0329**	DNA-directed RNA polymerase subunit beta'	Cytoplasmic	0	1	1	0	2
**VC0219**	50S ribosomal protein L33	Cytoplasmic	0	0	1	3	0
**VCA0033**	Transcriptional initiation protein Tat	Unknown	1	2	0	1	0
**VC2455**	Phosphate ABC transporter substrate-binding protein	Inner Membrane	1	0	0	0	3
**VC2435**	ADP-ribose pyrophosphatase	Cytoplasmic	2	0	1	0	1
**VC2195**	Flagellar basal-body rod protein FlgG	Extracellular	1	0	1	1	1
**VC1496**	Tail-specific protease	Inner Membrane	1	0	0	0	3
**VC1043**	Long-chain fatty acid transport protein	Outer Membrane	0	2	0	1	1
**VC0910**	PTS system, trehalose-specific IIBC component	Inner Membrane	2	1	0	1	0
**VC0841**	Accessory colonization factor AcfC	Unknown	2	1	0	0	1
**VC0550**	Oxaloacetate decarboxylase, alpha subunit	Cytoplasmic	1	1	0	0	2

**VCA0123**	Type VI secretion system spike protein VgrG3	Cytoplasmic	0	2	1	0	0
**VC2596**	50S ribosomal protein L3	Cytoplasmic	0	1	1	1	0
**VC2492**	3-isopropylmalate dehydratase large subunit	Cytoplasmic	0	0	1	0	2
**VC0941**	Serine hydroxymethyltransferase 1	Cytoplasmic	0	1	0	1	1
**VC0487**	Glutamine--fructose-6-phosphate aminotransferase [isomerizing]	Cytoplasmic	0	0	0	1	2
**VC0397**	Single-stranded DNA-binding protein	Cytoplasmic	0	0	0	2	1
**VCA0722**	Uncharacterized protein	Unknown	0	1	0	1	1
**VCA0563**	NAD(P) transhydrogenase subunit alpha	Inner Membrane	1	0	1	0	1
**VC2684**	Bifunctional aspartokinase/homoserine dehydrogenase	Cytoplasmic	0	0	0	1	2
**VC2420**	Flavodoxin	Unknown	1	0	2	0	0
**VC2414**	Pyruvate dehydrogenase E1 component	Cytoplasmic	0	1	0	2	0
**VC2280**	Putative ATP-dependent zinc protease domain-containing protein	Cytoplasmic	1	2	0	0	0
**VC2084**	Succinate--CoA ligase [ADP-forming] subunit alpha	Cytoplasmic	2	0	0	1	0
**VC2013**	PTS system glucose-specific EIICB component	Inner Membrane	2	1	0	0	0
**VC1964**	Tim44-like domain-containing protein	Inner Membrane	1	0	0	0	2
**VC1601**	Uncharacterized protein	Inner Membrane	1	0	0	0	2
**VC1267**	DUF1513 domain-containing protein	Inner Membrane	1	0	0	1	1
**VC1207**	DUF3187 family protein	Outer Membrane	2	0	0	0	1
**VC0200**	Iron(III) compound receptor	Outer Membrane	0	0	1	1	1
**VC0041**	DUF3157 domain-containing protein	Unknown	0	0	1	1	1
**VCA0740**	UPF0502 protein VC_A0740	Cytoplasmic	1	1	0	0	0
**VC2583**	30S ribosomal protein S14	Cytoplasmic	0	1	1	0	0
**VC2581**	50S ribosomal protein L6	Cytoplasmic	1	0	1	0	0
**VC2463**	Elongation factor 4	Inner Membrane	1	1	0	0	0
**VC2249**	3-hydroxyacyl-[acyl-carrier-protein] dehydratase FabZ	Cytoplasmic	1	1	0	0	0
**VC0468**	Glutathione synthetase	Cytoplasmic	0	0	0	1	1
**VCA1054**	NAD(P)-binding domain-containing protein	Unknown	1	0	1	0	0
**VCA1027**	Maltose operon periplasmic protein, putative	Periplasmic	0	0	0	1	1
**VCA0212**	Sugar ABC transporter ATPase	Cytoplasmic	1	0	0	0	1
**VCA0125**	DUF3012 domain-containing protein	Unknown	0	0	0	1	1
**VC2744**	50S ribosomal subunit assembly factor BipA	Inner Membrane	1	0	0	0	1
**VC2341**	Long-chain-fatty-acid--CoA ligase, putative	Cytoplasmic	1	0	0	1	0
**VC1896**	Uncharacterized protein	Inner Membrane	1	1	0	0	0
**VC1776**	N-acetylneuraminate lyase, putative	Cytoplasmic	1	1	0	0	0
**VC1555**	YhcH/YjgK/YiaL family protein	Cytoplasmic	0	0	1	0	1
**VC1249**	Glycine cleavage system transcriptional repressor	Inner Membrane	0	0	1	1	0
**VC1031**	Inosine monophosphate dehydrogenase-related protein	Unknown	1	0	0	1	0
**VC0457**	DUF4426 domain-containing protein	Unknown	0	0	0	1	1
**VC0392**	Aminotransferase, class V	Cytoplasmic	1	0	0	1	0
