# Supplementary material for: Outer membrane vesicles and the outer membrane protein OmpU govern Vibrio cholerae biofilm matrix assembly
Source: mBio. 2024 Jan 11;15(2):e03304-23. doi: 10.1128/mbio.03304-23 (PMC10865864; doi:10.1128/mbio.03304-23)
Supplement: Table S2 — OMV proteome. [file mbio.03304-23-s0003.pdf]

Supplementary Table 2. List of all proteins identified in OMV proteome

Label	Description	PsortB	Total spectral counts				
			O1	O2	O3	O4	O5
**VC0633**	Outer membrane protein U	Outer Membrane	2874	4464	5255	4865	6261
**VC1888**	Hemolysin-related protein	Extracellular	1659	916	846	698	703
**VC2211**	Vibriobactin receptor	Outer Membrane	232	446	879	821	1470
**VC1854**	Porin, putative	Outer Membrane	398	663	761	881	983
**VCA0738**	Conjugal transfer protein TraF	Unknown	419	484	740	772	847
**VC0930**	Hemolysin-related protein	Extracellular	894	484	468	375	342
**VC0475**	Iron-regulated outer membrane virulence protein	Outer Membrane	161	407	382	381	984
**VC1154**	Uncharacterized protein	Unknown	134	278	434	518	875
**VCA1028**	Maltoporin	Outer Membrane	150	272	443	518	729
**VCA0223**	Pre-pro-metalloprotease PrtV	Extracellular	487	219	441	416	334
**VC1894**	Penicillin-binding protein activator LpoB	Unknown	61	182	267	317	698
**VC1318**	Outer membrane protein OmpV	Outer Membrane	92	206	359	439	420
**VCA0568**	Conjugal transfer protein TraF	Outer Membrane	191	154	314	336	369
**VCA0576**	Heme transport protein HutA	Outer Membrane	112	371	161	242	409
**VC2213**	Outer membrane protein OmpA	Outer Membrane	65	290	208	248	460
**VCA0865**	Hemagglutinin/proteinase	Extracellular	317	100	251	235	183
**VCA0863**	Lysophospholipase VolA	Unknown	98	155	218	227	310
**VC0156**	Vitamin B12 transporter BtuB	Outer Membrane	62	165	191	202	287
**VC1485**	DUF3466 family protein	Unknown	88	128	174	168	255
**VC1384**	Outer membrane protein beta-barrel domain-containing protein	Unknown	71	97	152	146	160
**VC2305**	Outer membrane protein OmpK	Outer Membrane	50	80	102	131	260
**VC2174**	5'-nucleotidase	Periplasmic	47	206	73	65	226
**VC0972**	Porin, putative	Outer Membrane	57	101	115	102	225
**VC2252**	Outer membrane protein assembly factor BamA	Outer Membrane	38	75	87	106	172
**VC1836**	Tol-Pal system protein TolB	Periplasmic	28	96	77	90	171
**VC2436**	Outer membrane protein TolC	Outer Membrane	35	94	47	88	191
**VC1621**	Agglutination protein	Outer Membrane	22	115	40	60	177
**VC0446**	LPS-assembly protein LptD	Outer Membrane	32	94	84	79	112
**VC0146**	Ribosomal RNA small subunit methyltransferase D	Cytoplasmic	46	54	105	119	69
**VCA0867**	Outer membrane protein W	Outer Membrane	10	45	60	92	153
**VC0381**	Uncharacterized protein	Unknown	29	43	63	73	147
**VC0935**	Capsular polysaccharide synthesis enzyme CpsB	Outer Membrane	26	58	67	77	125
**VC2002**	DUF2860 domain-containing protein	Outer Membrane	49	46	74	74	89
**VCA0625**	TonB receptor-related protein	Outer Membrane	23	48	65	63	121
**VC2011**	OMP_b-brl domain-containing protein	Inner Membrane	16	48	43	71	110
**VC1329**	Opacity protein-related protein	Outer Membrane	24	39	52	61	100
**VC0200**	Iron(III) compound receptor	Outer Membrane	16	49	55	53	78
**VCA0813**	Aminopeptidase	Extracellular	122	13	32	23	19
**VC1867**	Lipid A deacylase LpxR family protein	Unknown	23	40	34	39	69
**VCA0862**	Long-chain fatty acid transport protein	Outer Membrane	22	47	38	37	60
**VC1895**	DUF1425 domain-containing protein	Unknown	23	40	38	29	69
**VC2298**	Lipoprotein, putative	Unknown	1	36	24	37	87
**VCA0734**	OMP_b-brl domain-containing protein	Unknown	16	29	43	36	60
**VCA0594**	Hemolysin	Unknown	2	32	31	47	71

**VC2456**	Uncharacterized protein	Outer Membrane	24	32	35	34	56
**VC0942**	Lipoprotein	Unknown	1	46	40	46	48
**VC1362**	Amino acid ABC transporter, periplasmic amino acid-binding protein	Periplasmic	14	48	42	29	45
**VC1091**	Oligopeptide ABC transporter, periplasmic oligopeptide-binding protein	Periplasmic	2	56	24	19	72
**VC1835**	Peptidoglycan-associated lipoprotein	Outer Membrane	8	23	19	21	52
**VC1207**	DUF3187 family protein	Outer Membrane	16	26	22	28	28
**VC0171**	Peptide ABC transporter, periplasmic peptide-binding protein	Periplasmic	11	16	20	36	37
**VC2142**	Flagellin B	Extracellular	0	25	16	25	48
**VC1042**	Long-chain fatty acid transport protein	Outer Membrane	7	18	27	25	30
**VC0928**	RbmA protein	Unknown	35	63	4	5	0
**VC2197**	Flagellar hook protein FlgE	Extracellular	2	25	10	13	56
**VC2168**	DUF2066 domain-containing protein	Unknown	0	17	7	16	66
**VCA0812**	Leucine aminopeptidase-related protein	Extracellular	11	12	34	17	25
**VC0581**	Penicillin-binding protein activator LpoA	Inner Membrane	1	28	8	7	51
**VC0608**	Iron(III) ABC transporter, periplasmic iron-compound-binding protein	Periplasmic	1	12	12	13	57
**VCA0559**	Solitary outer membrane autotransporter beta-barrel domain-containing protein	Unknown	9	15	21	27	21
**VCA0064**	TonB system receptor, putative	Outer Membrane	12	15	23	10	30
**VC0954**	LPS-assembly lipoprotein LptE	Outer Membrane	4	19	22	16	26
**VC2187**	Flagellin C	Extracellular	0	15	9	13	49
**VCA0975**	endopeptidase La	Cytoplasmic	32	15	18	12	7
**VC2143**	Flagellin D	Extracellular	3	12	16	12	38
**VC0755**	Peptidase B	Cytoplasmic	18	36	6	9	6
**VC1043**	Long-chain fatty acid transport protein	Outer Membrane	9	18	13	13	21
**VC1703**	Uncharacterized protein	Unknown	0	19	9	17	24
**VC1064**	Lipoprotein-related protein	Unknown	1	13	8	8	31
**VCA0877**	Hydrolase, putative	Unknown	4	13	15	21	7
**VC2188**	Flagellin A	Extracellular	0	8	7	10	32
**VCA0573**	DamX-related protein	Periplasmic	1	18	5	13	15
**VC2299**	Peptidyl-prolyl cis-trans isomerase	Periplasmic	5	10	10	13	14
**VC1987**	Outer membrane lipoprotein Slp, putative	Outer Membrane	1	14	5	11	19
**VC2156**	Outer membrane protein assembly factor BamC	Outer Membrane	0	13	2	4	30
**VC1663**	Heat shock protein HslJ	Unknown	0	10	1	0	38
**VC1200**	Trypsin, putative	Unknown	0	2	13	9	25
**VC2198**	Basal-body rod modification protein FlgD	Extracellular	0	6	2	6	32
**VCA0219**	Hemolysin	Extracellular	20	1	14	7	0
**VC2141**	Protein FlaG	Unknown	1	13	5	9	12
**VC2204**	Negative regulator of flagellin synthesis	Unknown	0	8	4	9	19
**VC0402**	MSHA biogenesis protein MshL	Outer Membrane	6	10	5	6	13
**VC1896**	Uncharacterized protein	Inner Membrane	1	10	11	4	11
**VC0578**	Hemolysin, putative	Periplasmic	0	6	7	13	10
**VC0554**	Protease, insulinase family/protease, insulinase family	Unknown	0	9	5	8	14
**VC2200**	Flagellar basal body rod protein FlgB	Periplasmic	0	6	4	9	16
**VC0844**	Accessory colonization factor AcfA	Outer Membrane	3	7	7	6	11
**VC0762**	Outer membrane protein assembly factor BamB	Outer Membrane	0	21	1	2	9
**VCA1027**	Maltose operon periplasmic protein, putative	Periplasmic	0	7	3	5	18

**VC2251**	Chaperone protein Skp	Periplasmic	0	10	3	9	9
**VC2196**	Flagellar basal-body rod protein FlgF	Periplasmic	0	6	4	7	13
**VC1496**	Tail-specific protease	Inner Membrane	0	8	4	6	12
**VC2764**	ATP synthase subunit beta	Cytoplasmic	4	9	10	4	2
**VC0905**	Probable D-methionine-binding lipoprotein MetQ	Inner Membrane	3	17	4	3	2
**VC2128**	Flagellar hook-length control protein FliK, putative	Extracellular	0	8	5	7	9
**VC1195**	Lipoprotein, putative	Outer Membrane	0	4	1	2	21
**VCA0581**	Peptidase M48 domain-containing protein	Unknown	0	12	1	2	12
**VC1606**	Outer membrane protein TolC	Unknown	2	9	3	7	6
**VC1425**	Putrescine-binding periplasmic protein	Periplasmic	0	12	4	1	10
**VC0533**	Lipoprotein NlpD	Outer Membrane	0	4	2	5	15
**VCA0981**	Solute-binding protein family 3/N-terminal domain-containing protein	Unknown	4	1	5	14	1
**VC2550**	YtfJ family protein	Inner Membrane	2	6	5	5	6
**VC1887**	Peptidoglycan-binding protein CsiV	Unknown	0	7	6	3	7
**VC1622**	Outer membrane protein, putative	Outer Membrane	0	6	6	4	7
**VC2455**	Phosphate ABC transporter substrate-binding protein	Inner Membrane	0	7	2	2	10
**VC0132**	Uncharacterized protein	Unknown	0	4	2	6	9
**VCA0195**	OMP_b-brl domain-containing protein	Outer Membrane	0	11	2	2	5
**VC1834**	Cell division coordinator CpoB	Unknown	0	3	1	2	14
**VC0430**	Immunogenic protein	Unknown	0	3	2	3	12
**VC0274**	DUF1481 domain-containing protein	Unknown	0	7	2	3	8
**VCA0037**	Copper chaperone PCu(A)C	Unknown	1	3	6	6	3
**VC1929**	C4-dicarboxylate-binding periplasmic protein DctP	Periplasmic	0	3	0	0	15
**VCA0759**	Arginine ABC transporter, periplasmic arginine-binding protein	Periplasmic	0	10	2	2	4
**VCA0459**	Lipoprotein	Unknown	0	5	1	3	9
**VC2527**	Lipopolysaccharide export system protein LptA	Unknown	0	7	1	2	8
**VC0157**	Alkaline serine protease	Extracellular	8	4	3	1	2
**VC2144**	Flagellin E	Extracellular	0	3	3	4	7
**VC2140**	Flagellar hook-associated protein 2	Extracellular	0	5	1	5	5
**VC0708**	Outer membrane protein assembly factor BamD	Outer Membrane	0	6	1	2	7
**VC2378**	DUF1499 domain-containing protein	Unknown	1	2	4	1	8
**VC1334**	Tricarboxylic transport TctC	Periplasmic	0	4	2	2	8
**VCA0058**	LysM domain-containing protein	Unknown	0	5	1	2	7
**VC1267**	DUF1513 domain-containing protein	Inner Membrane	1	2	3	2	7
**VC0688**	DUF2799 domain-containing protein	Unknown	0	1	1	2	11
**VC0362**	Elongation factor Tu-B	Cytoplasmic	1	8	2	1	2
**VC0409**	MSHA pilin protein MshA	Unknown	1	2	1	1	9
**VCA0106**	Sel1 repeat family protein	Cytoplasmic	0	4	2	3	4
**VC2662**	PhaC PHA synthase	Unknown	0	6	1	1	5
**VC0973**	VvgS protein	Unknown	0	6	0	2	5
**VC2249**	3-hydroxyacyl-[acyl-carrier-protein] dehydratase FabZ	Cytoplasmic	0	5	1	2	4
**VC0414**	DUF6701 domain-containing protein	Unknown	0	4	7	0	1
**VCA1008**	Outer membrane protein, putative	Outer Membrane	0	1	3	5	2
**VC2199**	Flagellar basal-body rod protein FlgC	Periplasmic	0	1	1	3	6
**VC0194**	Glutathione hydrolase proenzyme	Periplasmic	0	1	1	1	8
**VC0480**	Small-conductance mechanosensitive channel	Inner Membrane	1	9	0	0	0
**VC2195**	Flagellar basal-body rod protein FlgG	Extracellular	0	2	2	2	3

**VC1424**	Putrescine-binding periplasmic protein	Periplasmic	0	5	1	1	2
**VC2664**	Chaperonin GroEL 1	Cytoplasmic	0	6	0	0	2
**VC0432**	Malate dehydrogenase	Unknown	0	2	2	1	3
**VC0648**	Lipoprotein NlpI	Inner Membrane	0	2	1	1	4
**VCA0317**	Outer membrane lipoprotein Blc	Outer Membrane	0	1	1	1	4
**VC1560**	Catalase-peroxidase	Cytoplasmic	0	5	1	0	1
**VC0328**	DNA-directed RNA polymerase subunit beta	Cytoplasmic	2	4	1	0	0
**VCA0685**	Iron(III) ABC transporter, periplasmic iron-compound-binding protein	Periplasmic	1	1	2	1	2
**VCA0514**	Putative ATP-dependent zinc protease domain-containing protein	Unknown	0	5	1	1	0
**VC2191**	Flagellar hook-associated protein 1	Extracellular	0	2	0	2	3
**VC0450**	Membrane-bound lytic murein transglycosylase C	Cytoplasmic	0	3	1	1	2
**VC0851**	Outer membrane protein assembly factor BamE	Outer Membrane	0	1	0	2	3
**VC0341**	Oligoribonuclease	Cytoplasmic	0	1	1	1	3
**VCA0139**	DUF3316 domain-containing protein	Unknown	0	5	0	0	1
**VCA0059**	Major outer membrane lipoprotein Lpp	Outer Membrane	0	2	0	0	4
**VC2190**	Flagellar hook-associated protein FlgL	Extracellular	0	2	1	0	3
**VC1101**	Uncharacterized protein	Unknown	0	2	0	1	3
**VC1107**	Outer-membrane lipoprotein carrier protein	Periplasmic	0	1	1	1	2
**VC2181**	Outer-membrane lipoprotein LolB	Unknown	0	4	0	0	1
**VC1585**	Catalase	Periplasmic	0	3	1	0	1
**VC0361**	Elongation factor G 1	Cytoplasmic	1	1	1	2	0
**VCA0552**	Fatty acid cis/trans isomerase	Unknown	0	1	0	1	3
**VCA0227**	Iron(III) ABC transporter, periplasmic iron-compound-binding protein	Periplasmic	0	2	0	0	3
**VC2562**	2`,3`-cyclic-nucleotide 2`-phosphodiesterase	Periplasmic	0	1	1	1	2
**VC1638**	DNA-binding response regulator	Cytoplasmic	0	0	0	2	3
**VC0964**	PTS system, glucose-specific IIA component	Cytoplasmic	0	0	2	0	3
**VC2733**	Secretin GspD	Outer Membrane	0	2	1	1	0
**VC0948**	Endolytic peptidoglycan transglycosylase RlpA	Unknown	0	1	0	3	0
**VC0394**	UvrABC system protein A	Cytoplasmic	0	3	0	0	1
**VCA0722**	Uncharacterized protein	Unknown	0	0	2	1	1
**VCA0113**	Type VI secretion system lipoprotein TssJ	Unknown	0	2	0	2	0
**VC2517**	Phospholipid-binding protein MlaC	Unknown	0	2	0	0	2
**VC2341**	Long-chain-fatty-acid--CoA ligase, putative	Cytoplasmic	0	3	1	0	0
**VC1583**	Superoxide dismutase [Cu-Zn]	Periplasmic	0	0	1	1	2
**VC1266**	Imelysin-like domain-containing protein	Unknown	0	1	0	0	3
**VC0976**	Protein QmcA	Unknown	0	1	0	0	3
**VCA0539**	UPF0312 protein VC_A0539	Unknown	0	2	0	0	1
**VCA0197**	GMP reductase	Cytoplasmic	1	1	0	0	1
**VC2164**	Putative beta-barrel assembly-enhancing protease	Unknown	0	1	0	0	2
**VCA0900**	Metallo-beta-lactamase domain-containing protein	Unknown	0	2	0	0	1
**VCA0829**	Acetyl-CoA synthase	Cytoplasmic	0	2	0	1	0
**VCA0563**	NAD(P) transhydrogenase subunit alpha	Inner Membrane	1	2	0	0	0
**VCA0125**	DUF3012 domain-containing protein	Unknown	0	0	2	0	1
**VC2084**	Succinate--CoA ligase [ADP-forming] subunit alpha	Cytoplasmic	0	2	0	0	1
**VC1872**	PrkA AAA domain-containing protein	Cytoplasmic	1	2	0	0	0

**VC1863**	Amino acid ABC transporter, periplasmic amino acid-binding protein	Periplasmic	0	2	0	1	0
**VC1492**	glutamate dehydrogenase	Cytoplasmic	0	0	2	0	1
**VC1325**	Autoinducer 2-binding periplasmic protein LuxP	Periplasmic	0	1	0	0	2
**VC1162**	Putative ATP-dependent zinc protease domain-containing protein	Cytoplasmic	0	1	0	0	2
**VC2574**	30S ribosomal protein S13	Cytoplasmic	0	1	1	0	0
**VC2350**	Deoxyribose-phosphate aldolase	Cytoplasmic	0	0	1	1	0
**VC0445**	Chaperone SurA	Periplasmic	0	1	0	0	1
**VC2744**	50S ribosomal subunit assembly factor BipA	Inner Membrane	1	1	0	0	0
**VC2004**	Haem-binding uptake Tiki superfamily ChaN domain-containing protein	Unknown	1	1	0	0	0
**VC1952**	Chitinase	Extracellular	0	0	1	0	1
**VC1950**	trimethylamine-N-oxide reductase	Periplasmic	0	0	1	0	1
**VC1755**	ABC-type transport auxiliary lipoprotein component domain-containing protein	Unknown	0	1	0	0	1
**VC0174**	SPOR domain-containing protein	Periplasmic	0	1	0	1	0
