# Supplementary material for: Outer membrane vesicles and the outer membrane protein OmpU govern Vibrio cholerae biofilm matrix assembly
Source: mBio. 2024 Jan 11;15(2):e03304-23. doi: 10.1128/mbio.03304-23 (PMC10865864; doi:10.1128/mbio.03304-23)
Supplement: Table S3 — ompU-WT proteome comparison. [file mbio.03304-23-s0004.pdf]

Supplementary Table 3. Proteins, significantly up- and down-regulated in Δ*ompU* compared to rugose strain.

Gene	Description	Fold change (log2)
**Proteins significantly up-regulated in Δ*ompU***		
VC_2662	Hypothetical protein	2.543
VC_A0713	Hypothetical protein	2.3357
VC_2213	Outer membrane protein A precursor	2.3031
VC_2216	CopG protein	1.9661
VC_A0139	Acyl-CoA synthetase	1.8961
VC_A0707	Phosphoglycerate transporter protein PgtP	1.6996
VC_1318	Outer membrane protein OmpV	1.6253
VC_0935	Capsular polysaccharide synthesis enzyme VpsM	1.5661
VC_0972	Chitin outer membrane porin	1.5632
VC_1854	Outer membrane protein OmpT	1.5583
VC_0437	GTP-binding protein Obg	1.4761
VC_2002	Hypothetical protein	1.4356
VC_0475	TonB-dependent receptor	1.3718
VC_0157	Alkaline serine exoprotease A precursor, IvaP	1.3707
VC_0486	Transcriptional regulator of glmS gene, DeoR family	1.3545
VC_1834	Cell division coordinator CpoB	1.2497
VC_0487	Glutamine—fructose-6-phosphate aminotransferase	1.206
VC_0296	Biotin carboxyl carrier protein of acetyl-CoA carboxylase	1.1966
VC_0156	Outer membrane vitamin B12 receptor BtuB	1.1852
VC_1835	Tol-Pal system peptidoglycan-associated lipoprotein PAL	1.1596
VC_1965	Transcriptional regulator, AcrR family	1.1362
VC_1966	Lysophospholipid transporter LplT/2-acylglycerophosphoethanolamine acyltransferase	1.0881
VC_A0952	Transcriptional regulator, VpsT	1.058
VC_A0962	Hypothetical protein	1.0405
VC_A0223	Metalloprotease, putative zinc-binding domain, PrtV	1.0144
VC_A0568	TraF-related protein	1.0129
VC_1895	YcfL protein: an OM lipoprotein that is part of a salvage cluster	1.0072
**Proteins significantly down-regulated in Δ*ompU***		
VC_0633	Outer membrane porin, OmpU	-4.8762
VC_0911	Trehalose-6-phosphate hydrolase	-1.4309
VC_2240	Phenolic acid decarboxylase	-1.4162
VC_A0551	Hypothetical protein	-1.3288
VC_0566	Outer membrane stress sensor protein DegQ, serine protease	-1.2519
VC_A0935	Hypothetical protein	-1.2307
VC_0034	Periplasmic thiol:disulfide interchange protein DsbA	-1.2149
VC_1611	Homoserine O-succinyltransferase	-1.0731
VC_A0032	Hypothetical protein	-1.0356
