# Supplementary material for: Outer membrane vesicles and the outer membrane protein OmpU govern Vibrio cholerae biofilm matrix assembly
Source: mBio. 2024 Jan 11;15(2):e03304-23. doi: 10.1128/mbio.03304-23 (PMC10865864; doi:10.1128/mbio.03304-23)
Supplement: Table S4 — Strains and plasmids. [file mbio.03304-23-s0005.pdf]

Supplementary Table 4. Strains and plasmid list

Strain or plasmid	Relevant genotype	Source
***E. coli* strains**		
DH5-α λ *pir*	F' *endA1 hsdR17 supE44 thi-1 recA1 gyrA96 relA1* Δ(*argF-lacZYA*) U169(Φ80*lac*D*M15*) λ *pir*	BCCM Gene corner
CC118 λ *pir*	Δ(*ara-leu*) *araD* Δ*lac*X74 *galE galK phoA20 thi-1 rpsE rpoB argE*(*Am*) *recA1* λ *pir*	(1)
S17-1 λ *pir*	Tp^r Sm^r *recA thi pro* rK^- mK^+ RP4::2-Tc::MuKm Tn7 λ *pir*	(2)
***V. cholerae* strains**		
FY_Vc_2	*Vibrio cholerae* O1 El Tor A1552, Rugose variant, Rif^r	(3)
FY_Vc_7044	FY_Vc_2 Δ*rbmA* (VC0928)	This work
FY_Vc_686	FY_Vc_2 Δ*rbmC* (VC0930)	(4)
FY_Vc_1367	FY_Vc_2 Δ*bap1* (VC1888)	(4)
FY_Vc_4329	FY_Vc_2 Δ*rbmA* Δ*rbmC* Δ*bap1*	(5)
FY_Vc_4327	FY_Vc_2 Δ*vps-I* Δ*vps-II*	(6)
FY_Vc_9251	FY_Vc_2 Δ*toxR*	This work
FY_Vc_17473	FY_Vc_9251 pMMB67.EH-*ompU*	This work
FY_Vc_13387	FY_Vc_2 Δ*ompU* (VC0633)	This work
FY_Vc_16739	FY_Vc_2 Δ*ompT* (VC1854)	This work
FY_Vc_13385	FY_Vc_2 Δ*ompA* (VC2213)	This work
FY_Vc_16843	FY_Vc_2 Δ*ompV* (VC1318)	This work
FY_Vc_13724	FY_Vc_13387 Tn7::Pnat-*ompU*	This work
FY_Vc_240	FY_Vc_2 Tn7-GFP	(7)
FY_Vc_16827	FY_Vc_4327 Δ*ompU*	This work
FY_Vc_16821	FY_Vc_7044 Δ*ompU*	This work
FY_Vc_17026	FY_Vc_686 Δ*ompU*	This work
FY_Vc_17028	FY_Vc_1367 Δ*ompU*	This work
FY_Vc_16823	FY_Vc_4329 Δ*ompU*	This work
FY_Vc_8970	Δ*ctxAB* Δ*rbmA* Δ*rbmC bap1*-3xHA	This work
FY_Vc_8966	Δ*ctxAB* Δ*rbmA* Δ*bap1 rbmC*-3xFLAG	This work
FY_Vc_9441	Δ*ctxAB* Δ*rbmC* Δ*bap1 rbmA*-Myc	This work
FY_Vc_17245	FY_Vc_13387 ΔVC2662	This work
FY_Vc_17262	FY_Vc_13387 ΔVCA0713	This work
FY_Vc_9944	FY_Vc_2 Δ*degP* (VC0566)	This work
FY_Vc_6228	FY_Vc_4329 Tn7-GFP	This work
FY_Vc_6226	FY_Vc_4327 Tn7-GFP	(6)
FY_Vc_10559	Δ*ctxAB* Δ*rbmA* Δ*rbmC* Δ*bap1* Δ*vps-I* Δ*vps-II*	This work
**Plasmids**		
pGP704-*sacB*28	pGP704 derivative, *mob*/*oriT sacB*, Amp^r	G. Schoolnik
pFY_1277	pGP704-*sacB*28::Δ*rbmA*, Amp^r	This work
pFY_5536	pGP704-*sacB*28::Δ*ompU*, Amp^r	This work
pFY_5542	pGP704-*sacB*28::Δ*ompT*, Amp^r	This work
pFY_5535	pGP704-*sacB*28::Δ*ompA*, Amp^r	This work

pFY_5654	pGP704-*sacB*28::Δ*ompV*, Amp^r	This work
pFY_4306	pGP704-*sacB*28::Δ*toxR*, Amp^r	This work
pFY_4832	pGP704-*sacB*28::ΔVC2662, Amp^r	This work
pFY_6949	pGP704-*sacB*28::ΔVCA0713, Amp^r	This work
pFY_295	pGP704-*sacB*28::Δ*ctxAB*, Amp^r	(5)
pFY_1057	pGP704-*sacB*28::*rbmA*-Myc, Amp^r	(5)
pFY_1093	pGP704-*sacB*28::*rbmC*-3xFLAG, Amp^r	(5)
pFY_1159	pGP704-*sacB*28::*bap1*-3xHA, Amp^r	(5)
pFY_1303 (pMMB67.EH)	Expression vector harboring *lacI* and containing a Ptac promoter, Amp^r	(8)
pFY_7013	pMMB67.EH-*ompU*, Amp^r	This work
pMCM11	pGP704::mTn7-*gfp*, Gm^r Amp^r	M. Miller and G. Schoolnik
pFY_720	pGP704-Tn7, Gm^r Amp^r	(9)
pFY_5722	pGP704-Tn7::*ompU*	This work

REFERENCES

1. Herrero M, de Lorenzo V, Timmis KN. 1990. Transposon vectors containing non-antibiotic resistance selection markers for cloning and stable chromosomal insertion of foreign genes in gram-negative bacteria. J Bacteriol 172:6557-67.
2. de Lorenzo V, Timmis KN. 1994. Analysis and construction of stable phenotypes in gram-negative bacteria with Tn5- and Tn10-derived minitransposons. Methods Enzymol 235:386-405.
3. Yildiz FH, Schoolnik GK. 1999. *Vibrio cholerae* O1 El Tor: identification of a gene cluster required for the rugose colony type, exopolysaccharide production, chlorine resistance, and biofilm formation. Proc Natl Acad Sci U S A 96:4028-33.
4. Fong JC, Yildiz FH. 2007. The *rbmBCDEF* gene cluster modulates development of rugose colony morphology and biofilm formation in *Vibrio cholerae*. J Bacteriol 189:2319-30.
5. Berk V, Fong JC, Dempsey GT, Develioglu ON, Zhuang X, Liphardt J, Yildiz FH, Chu S. 2012. Molecular architecture and assembly principles of *Vibrio cholerae* biofilms. Science 337:236-9.
6. Fong JCN, Syed KA, Klose KE, Yildiz FH. 2010. Role of *Vibrio* polysaccharide (vps) genes in VPS production, biofilm formation and *Vibrio cholerae* pathogenesis. Microbiology (Reading) 156:2757-2769.
7. Beyhan S, Yildiz FH. 2007. Smooth to rugose phase variation in *Vibrio cholerae* can be mediated by a single nucleotide change that targets c-di-GMP signalling pathway. Mol Microbiol 63:995-1007.
8. Furste JP, Pansegrau W, Frank R, Blocker H, Scholz P, Bagdasarian M, Lanka E. 1986. Molecular cloning of the plasmid RP4 primase region in a multi-host-range *tacP* expression vector. Gene 48:119-31.
9. Lim B, Beyhan S, Meir J, Yildiz FH. 2006. Cyclic-diGMP signal transduction systems in *Vibrio cholerae*: modulation of rugosity and biofilm formation. Mol Microbiol 60:331-48.
